# Antibody Drug Conjugates for Cancer Therapy: From Metallodrugs to Nature-Inspired Payloads

**DOI:** 10.3390/ijms25168651

**Published:** 2024-08-08

**Authors:** Giovanni Tonon, Flavio Rizzolio, Fabiano Visentin, Thomas Scattolin

**Affiliations:** 1 Department of Molecular Sciences and Nanosystems, Università Ca’ Foscari Campus Scientifico, Via Torino 155, 30174 Venezia-Mestre, Italy; giovanni.tonon@unive.it (G.T.); flavio.rizzolio@unive.it (F.R.); 2 Pathology Unit, Department of Molecular Biology and Translational Research, Centro di Riferimento Oncologico di Aviano (CRO) IRCCS, Via Franco Gallini 2, 33081 Aviano, Italy; 3 Dipartimento di Scienze Chimiche, Università degli Studi di Padova, Via Marzolo 1, 35131 Padova, Italy

**Keywords:** antibody–drug conjugates, metallodrugs, natural compounds, cancer therapy

## Abstract

This review highlights significant advancements in antibody–drug conjugates (ADCs) equipped with metal-based and nature-inspired payloads, focusing on synthetic strategies for antibody conjugation. Traditional methods such us maleimide and succinimide conjugation and classical condensation reactions are prevalent for metallodrugs and natural compounds. However, emerging non-conventional strategies such as photoconjugation are gaining traction due to their milder conditions and, in an aspect which minimizes side reactions, selective formation of ADC. The review also summarizes the therapeutic and diagnostic properties of these ADCs, highlighting their enhanced selectivity and reduced side effects in cancer treatment compared to non-conjugated payloads. ADCs combine the specificity of monoclonal antibodies with the cytotoxicity of chemotherapy drugs, offering a targeted approach to the elimination of cancer cells while sparing healthy tissues. This targeted mechanism has demonstrated impressive clinical efficacy in various malignancies. Key future advancements include improved linker technology for enhanced stability and controlled release of cytotoxic agents, incorporation of novel, more potent, cytotoxic agents, and the identification of new cancer-specific antigens through genomic and proteomic technologies. ADCs are also expected to play a crucial role in combination therapies with immune checkpoint inhibitors, CAR-T cells, and small molecule inhibitors, leading to more durable and potentially curative outcomes. Ongoing research and clinical trials are expanding their capabilities, paving the way for more effective, safer, and personalized treatments, positioning ADCs as a cornerstone of modern medicine and offering new hope to patients.

## 1. Introduction

In the dynamic landscape of cancer therapy, antibody–drug conjugates (ADCs) stand out as a beacon of innovation, offering a multifaceted approach to targeting and treating malignancies with precision and efficacy [1,2,3]. These marvels of biotechnology marry the specificity of monoclonal antibodies (mAbs) with the potent cytotoxicity of chemotherapeutic agents, presenting a paradigm shift in the fight against cancer.

ADCs are the result of the strategic fusion of three critical components: the monoclonal antibody, the linker, and the cytotoxic payload. The monoclonal antibody serves as the guiding hand, homing in on specific antigens overexpressed on the surface of cancer cells while sparing healthy tissues [4,5,6]. This selective binding mechanism ensures that the cytotoxic payload is delivered exclusively to cancer cells, minimizing collateral damage to surrounding tissue and reducing systemic toxicity [1,2,3]. 

The linker, a molecular bridge connecting the antibody and the cytotoxic payload, plays a pivotal role in the controlled release of the therapeutic cargo [7]. Linkers can be engineered to exhibit different properties, such as stability in circulation and selective cleavage in the tumor microenvironment [8,9]. This flexibility allows for tailored release kinetics, optimizing the therapeutic window of ADCs and maximizing their efficacy.

Meanwhile, the cytotoxic payload represents the firepower of ADCs, capable of delivering a lethal blow to cancer cells upon release. These payloads encompass a spectrum of chemotherapeutic agents, ranging from traditional cytotoxic drugs to novel compounds with enhanced potency and unique mechanisms of action [10,11,12,13]. By leveraging the specificity of the antibody to target cancer cells, ADCs ensure that the cytotoxic payload reaches its intended destination, unleashing its anticancer potential with precision.

One of the most compelling aspects of ADCs is their ability to address the challenge of tumor heterogeneity, a hallmark of cancer that poses significant obstacles to treatment success [14,15,16,17,18]. By targeting specific antigens expressed on cancer cells, ADCs can effectively eradicate heterogeneous tumor populations, including primary tumors and metastatic lesions with distinct antigen profiles [1,2,3,19]. This targeted approach not only improves treatment efficacy but also reduces the likelihood of tumor resistance, a common criticism in conventional chemotherapy [20,21,22]. 

The clinical landscape of ADCs is rapidly evolving, with several agents having received regulatory approval for the treatment of various cancer types [23]. Examples include ado-trastuzumab emtansine (Kadcyla) for HER2-positive breast cancer [24], brentuximab vedotin (Adcetris) for Hodgkin lymphoma and systemic anaplastic large-cell lymphoma [25], and enfortumab vedotin (Padcev) for metastatic urothelial cancer [26]. These approvals underscore the amazing potential of ADCs in improving patient outcomes and reshaping the treatment paradigm for cancer.

However, challenges remain on the road to widespread adoption and optimization of ADCs in clinical practice. These include fine-tuning antibody specificity and affinity, optimizing linker design for enhanced stability and payload release kinetics, mitigating off-target toxicity, and overcoming mechanisms of resistance [1,2,3,19,27]. Addressing these challenges will require continued investment in research and development, as well as collaboration across interdisciplinary fields to unlock the full potential of ADCs in oncology.

In this contribution, we provide an overview of recent advancements and future perspectives in this important area of research, with a special focus on ADCs containing metal-based or natural-compound-based payloads.

Metal-based chemotherapeutic agents, which include platinum [28,29,30,31,32], ruthenium [33,34,35,36], gold [37,38,39,40,41], iron [42,43,44,45], palladium [46,47,48,49,50], and other transition metal complexes [51,52,53], have demonstrated remarkable efficacy against various types of cancer and continue to be a subject of intense research and clinical investigation. Platinum-based drugs such as cisplatin, carboplatin, and oxaliplatin are particularly important, being the most widely used metal-based chemotherapeutic agents in clinical practice [28,29,30,31]. Despite their potent cytotoxicity, platinum-based drugs are associated with significant side effects, including nephrotoxicity, neurotoxicity, and myelosuppression, which limit their clinical utility [54]. 

While many compounds containing metals other than platinum have demonstrated promising antitumor activity in both in vitro and in vivo models, only a very limited number of derivatives are in the final stages of clinical trials [55,56]. Despite offering a wider range of potential interactions with biotargets compared to purely organic compounds, metal complexes often react with other biological substrates besides the selected biotarget, thereby increasing their systemic toxicity [51]. 

For this reason, two main strategies are usually employed to improve the stability and selectivity of potential metallodrugs: (i) the use of ligands firmly anchored to the metal center (e.g., organometallic fragments and/or chelating ligands) [57,58,59], and (ii) conjugation to molecular targeting agents such as monoclonal antibodies [60], estrogen receptor antagonists [61,62], and immune checkpoint inhibitors [63,64]. The latter strategy will be examined in one of the upcoming chapters, focusing exclusively on conjugation with monoclonal antibodies.

As far as natural compounds are concerned, they have emerged as promising payloads in antibody–drug conjugates (ADCs), harnessing the therapeutic potential of plant-derived or naturally occurring substances for targeted cancer therapy [65,66,67]. The integration of these compounds into ADCs offers a unique avenue for delivering potent anticancer agents while minimizing systemic toxicity and off-target effects. 

The utilization of natural compounds as payloads in ADCs presents several advantages [65,66,67]. Firstly, these compounds often exhibit unique mechanisms of action and lower cross-resistance compared to conventional chemotherapeutic agents, offering new opportunities to overcome drug resistance. Secondly, natural compounds are generally well-tolerated and have favorable pharmacokinetic profiles, making them attractive candidates for incorporation into ADCs. Lastly, the diversity of natural compounds provides a rich source of potential payloads, allowing for the development of ADCs tailored to specific cancer types or molecular subtypes.

The most relevant examples include flavonoids, polyphenols, alkaloids, maytansinoids, auristatins, dolastatins, and molecules derived from microbial sources, such as bacterial or fungal toxins and microbial metabolites [65]. In this review, ADCs containing nature-inspired payloads reported in the literature over the past five years will be discussed in detail, examining the synthetic strategies employed, the types of linkers utilized, and the therapeutic effects observed by various authors.

## 2. Photosynthetic Approaches to Metal-Based ADCs

We have decided to begin this chemical journey by examining the most recent non-conventional synthetic approaches towards ADCs bearing metal-based drugs as payloads.

It should be remembered that traditional protein–small molecule conjugation techniques consist of methods that are highly successful but with some limitations. First, they are time-consuming and difficult to automate [68,69,70]. Second, they require multiple step reactions and, often, the chemical reagents used are incompatible with protein formulation buffers [71]. For example, conjugation of desferrioxamine B with ^89^Zr-mAbs requires a non-trivial pre-purification of the mAb from the formulation components [71,72].

In this context, Holland’s research group, working in Zurich, introduced an interesting approach for the synthesis of radiolabelled antibodies; called “photoradiosynthesis”, it exploits the activation of a photoactive substrate to generate electrophiles that react in situ with nucleophilic amino acid residues [73]. This technology allows the production of radiolabelled antibodies in a one-pot process starting from formulated antibodies (e.g., trastuzumab and onartuzumab), and it is characterized by the following: (i) rapid reaction kinetics (less than 10 min for both radiolabelling and conjugation), with reaction rates directly proportional to light intensity; (ii) high chemoselectivity; (iii) use of aerobic conditions and aqueous solutions in mild pH ranges (7–9) which are compatible with formulated buffers used to stabilize clinical-grade formulations of mAbs; (iv) negligible absorption by antibodies at the wavelengths used for the photoconjugation; and (v) ease in making the process fully automated. Moreover, the development of one-pot procedures allows researchers to obtain formulated products directly from formulated antibodies. 

Herein, we summarize the photoactive groups suitable for the conjugation of antibodies according to the classes described by Holland and co-workers [73]. 

**Scheme 1:**
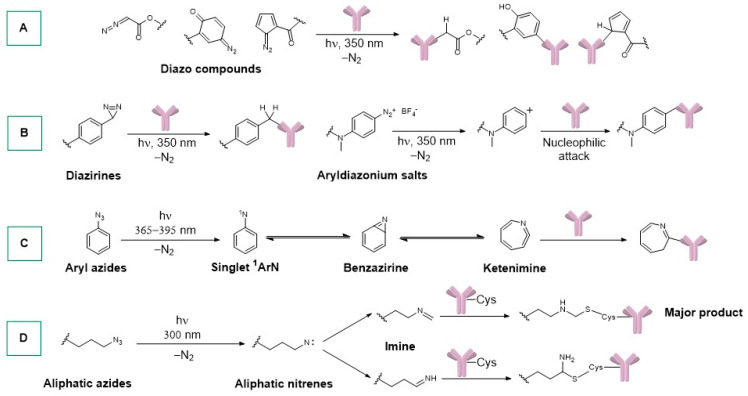
Photoactivation and conjugation of (**A**) diazo compounds, (**B**) diaziridines, aryldiazonium salts, and (**C**) aromatic and (**D**) aliphatic azides with proteins/antibodies.

**Diazo compounds** can be used for bioconjugation reactions, since, as a result of the irradiation, they can lose molecular nitrogen, leading to reactive carbenes (Scheme 1A). In order to avoid undesirable rearrangements, diazo compounds require the absence of β-hydrogen atoms and/or the presence aryl substituents. The reactivity of the intermediate carbene species allows the insertion into protein C-H bonds, resulting in a covalent conjugation. However, the use of diazo compounds does not guarantee the chemo- and regioselectivity of the process, and in some cases, the decomposition of reactants after dinitrogen loss was observed. In addition, for an efficient conjugation, a pre-association with the antibody is usually required.

**Aryl diazirines** were used by Smith and Knowles for the first time in 1973 [74]. Compared to diazocompounds, aryl diazirines exhibit increased thermal stability and can be easily synthesized. Moreover, they present efficient photoactivation properties by producing highly reactive singlet carbenes (Scheme 1B). Also in this case, the conjugation process is usually not chemo- and regioselective and requires pre-association with the antibody for an efficient conjugation. Aryl diazirines promote a 1,3-dipolar cycloaddition, being able to generate nitrile ylides that can react with an alkyne/alkene functionalized protein. Unfortunately, the formation of the final pyrroline derivatives requires a pre-functionalization of the protein, and the energy of the radiation (302 nm) can cause protein photodegradation.

**Diazonium salts** can be used since they are able to generate, by irradiation, a reactive carbenium ion (Scheme 1B). In this case, the bioconjugation reaction can be classified as a photoactivated nucleophilic aromatic substitution. Unfortunately, this method is, to the best of our knowledge, scarcely explored in the formation of ADCs containing metal-based payloads. 

**Aryl azides** can be irradiated to obtain interesting derivatives suitable for bioconjugation via the formation of an azepine compound (Scheme 1C). The first example of synthesis of an azepine ring by aryl azide photolysis was reported in 1966 [75]. The activation reaction affords a singlet nitrene by loss of dinitrogen; this intermediate can rearrange to a benzazirine bicycle and then to a ketenimine (dihydroazepine). This is the principal pathway at room temperature [76,77]. This type of derivative also enables the specific labelling of an antibody with amino acid residues. In this context, the Rousselot research group demonstrated the specific photolabelling between Tyr-49 residue of a mouse IgG1 mAb and an arylazidoestradiol derivative [78]. 

**Alkyl azides** are also suitable for bioconjugation reactions, exploiting, again, the extrusion of dinitrogen to obtain reactive aliphatic nitrenes [73]. The latter can easily rearrange to imine derivatives that react with nucleophilic residues of proteins; this method is chemoselective when sulfhydryl groups are present in the protein used (Scheme 1D). Notably, the first example of aliphatic azides used as a photolabelling reagent was reported in 1978 [79]. 

**Scheme 2:**
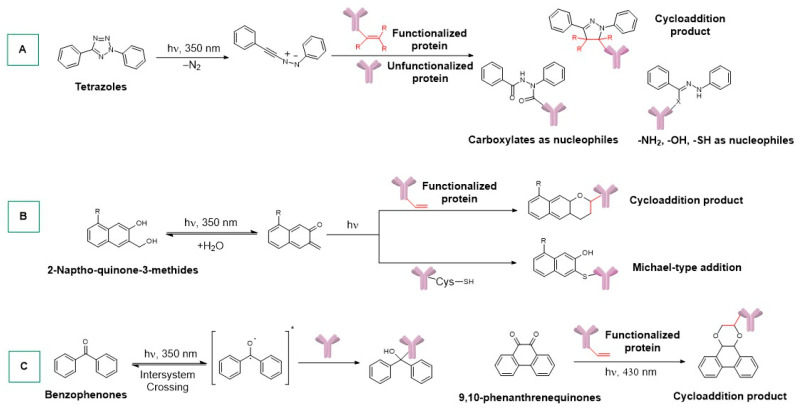
Photoactivation and conjugation of (**A**) tetrazoles, (**B**) 2-naphto-quinone-3-methides, (**C**) benzophenones, and phenanthrenequinones with proteins/antibodies.

**Tetrazoles** can be used for the photo-formation of nitrile imines, as reported for the first time by Huisgen and Sustmann [80]. Exploiting an alkene moiety of a functionalized protein, nitrile imines undergo 1,3-dipolar cycloaddition with the formation of the desired conjugation product (Scheme 2A) [73]. When unfunctionalized proteins are used, nitrile imines can react with carboxylate, amino, hydroxyl, and sulfhydryl groups (Scheme 2A). Unfortunately, the photoactivation of tetrazoles requires irradiation at 302 nm, which can potentially cause protein damage [73]. 

**Diels-Alder [4 + 2] cycloadditions** can be performed using 2-naphtoquinone-3-methides with proteins bearing vinyl groups, but the formation of the reactive diene species is reversible (Scheme 2B). Therefore, the reactive intermediate can be restored and react with nucleophiles like sulfhydryls in a Michael-type reaction. Alternative substrates include *ortho*-quinodimethanes that can undergo Diels–Alder cycloadditions with proteins pre-functionalized with a suitable dienophile [73]. 

**Benzophenone** photoexcitation can generate the activate molecule in a diradical triplet state (reversible reaction) that is reactive towards C-H insertion (Scheme 2C) [73]. In this case, the photoconjugation operates at λ > 330 nm (no protein damage), and the reaction requires the pre-association with the protein.

**9,10-Phenanthrenequinones** can be used in an interesting bioconjugation approach in the presence of an alkene-functionalized protein via a biradical [4 + 2] cycloaddition under visible light (Scheme 2C) [81]. The reaction also can be performed in live cells, and the presence of water is very well tolerated. 

Taking advantage of the methods described above, the synthesis of the conjugates of several protein/metal complexes, with interesting applications in theranostics, has recently been developed. Most of these ADCs contain lutetium, zirconium, and gallium as metal centers.

^177^Lutetium is a radiometal that decays through β^−^ emission of 176, 384, and 497 keV, and has a half-life of 6.6 days. Considering that the β^−^ emission of ^177^Lu has a penetration of ~2 mm in vivo, this metal is ideal for the irradiation of small tumors or metastases [82]. Moreover, the long half-life of ^177^Lu is useful for hospitals that do not have access to cyclotron facilities [82]. The main disadvantage of ^177^Lu concerns its inertness with respect to the formation of complexes with ligands such as DOTA; therefore, well-established/traditional methodologies cannot be applied. However, this issue can be overcome using a photosynthetic approach. 

**Scheme 3:**
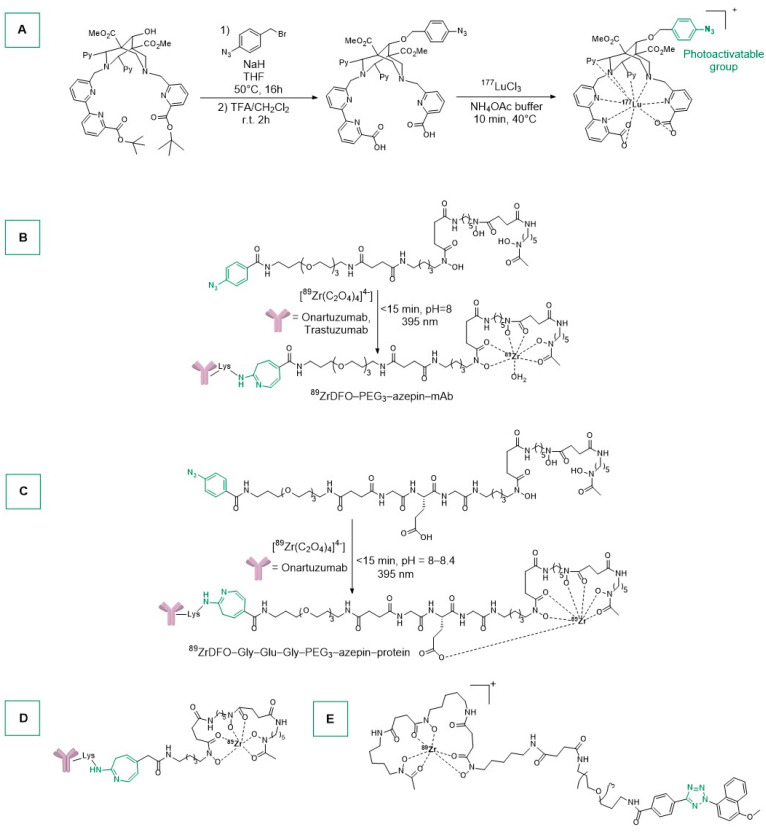
(**A**): Synthesis of the first ^177^Lu-bispidine conjugable complex, as reported by Holland. (**B**,**C**): ^89^Zr-DFO immunoconjugates reported by Holland. (**D**): ^89^Zr-immunoradiocomplex reported by Holland. (**E**): The ^89^Zr-immunoradiocomplex for molecular imaging bears a tetrazole moiety.

In this context, Holland and colleagues reported the synthesis of the first ^177^Lu-labelled mAb through a nonadentate bispidine ligand containing a photoactivatable ArN_3_ group (Scheme 3A) [83]. Notably, the complexation occurred under extremely mild conditions (40 °C, 10 min) [84,85,86]. L.E.D. irradiation (15 min, 23 °C, 395 nm) of a mixture of the complex and trastuzumab led to the desired ADC [^177^Lu-mAb], as confirmed by radio-instant Thin Layer Chromatography (radio-iTLC). The conjugation efficiency was estimated by SEC-chromatography to be 16%. After a spin-filtration purification, the [^177^Lu-mAb] was obtained with a radiochemical yield (RCY) of 12.5% and radiochemical purity (RCP) > 90%. Pharmacokinetics and tumor uptake were evaluated in female athymic nude mice bearing subcutaneous SKOV-3 tumors on the right flank. Two groups of mice were treated with high doses (8.2 MBq/mg) or low doses (0.406 MBq/mg, blocking group) of mAb-conjugate. The blocking group experiment consisted of adding a dose of the free antibody trastuzumab to saturate the available HER2/neu receptors for in vivo evaluation of [^177^Lu-mAb] specificity. Despite the different administered chemical doses, no differences were observed in the excretion rates between the two groups of mice. After 96 h post-injection, the accumulated radioactivity in SKOV-3 tumors was higher for the normal group than the blocking group (30 ± 11% ID/g vs. 15 ± 6% ID/g), confirming the specific targeting of HER2/neu by [^177^Lu-mAb].

^89^Zr, a radiometal with t_1/2_ = 78.4 h (β^+^), is currently under study for PET application, and is produced via proton-beam irradiation of commercially available ^89^Y solid metal foils [82]. The aqueous phase chemistry of Zr is limited to the oxidation state +4, and therefore, to avoid the precipitation of the insoluble Zr(OH)_4_, the use of chelating ligands like desferrioxamine B (DFO) is required [82]. Regarding PET application, ^89^Zr radiotracers remain radioactive for extended periods of time, and a typical dose for imaging HER2/*neu* expression in breast cancer patients is about 37 MBq/patient [82]. 

Deferoxamine B, a natural iron chelator [87] (siderophore) produced by *actinomycetes* [88], was photoconjugated (via phenylazide group) with onartuzumab and trastuzumab antibodies by Holland and co-workers after functionalization of the ligand with PEG linkers to improve the water solubility (Scheme 3B) [89]. It should be remembered that onartuzumab is a monoclonal antibody that binds to the human hepatocyte growth factor receptor c-MET [90,91]. In vivo tests on mouse models were conducted using 59–72 μg of ^89^ZrDFO-PEG_3_-azepin-onartuzumab per animal (normal dose) and 823–834 μg per animal (blocking group; to saturate human hepatocyte growth-factor receptor c-MET). The normal group was shown to have a higher accumulation of Zr radioactivity in the tumor than the control group. It should be noted that ^89^ZrDFO-PEG_3_-ArN_3_ and photolyzed ^89^ZrDFO-PEG_3_-azepin were rapidly removed from circulation, since no radioactivity in heart/blood was observed.

Other deferoxamine B-derived ligands were synthesized and conjugated to onartuzumab (Scheme 3C). The final products were characterized by radio-iTLC and size-exclusion chromatography and SEC-HPLC-coupled to a gel-filtration column [91]. The crude samples were purified by spin-filtration to remove the hydrolyzed by-products produced after photolysis of the PhN_3_ moiety. The zirconium bioconjugate complex was injected via the tail-vein in female athymic nude mice bearing subcutaneous MKN-45 human xenograft on the right shoulder. The blocking group received a 15-fold higher protein dose to saturate the c-MET receptor. The tumor uptake for the blocking group was 44% smaller than the normal one and biodistribution studies proved that 72 h after the injection, the accumulation of the Zr tracer in the tumor tissue was higher for the normal group. Moreover, low bone and background organ accumulation was noticed in both groups, proving that complexes with heptadentate ligands can be promising radiotracers for PET imaging [91]. 

A similar radioimmunoconjugate was reported by the same group using two different synthetic procedures [92]. The two-step procedure consisted in the conjugation between the ligand and the antibody and, in a second phase, the introduction of the radiometal. Specifically, the ligand DFO-ArN_3_ was conjugated to trastuzumab at room temperature within 25 min and then purified by SEC. The immunoconjugate was radiolabeled with ^89^Zr, starting from [^89^Zr(C_2_O_4_)_4_]^2−^ precursor and incubating the solutions at room temperature for 1 h (Scheme 3D). Afterwards, the reaction was quenched by adding small aliquots of EDTA. The authors reported also a one-pot procedure in which [^89^Zr(C_2_O_4_)_4_]^2−^ (aq.) (the ligand) and trastuzumab were reacted in water (pH = 8–9) at room temperature. The irradiation at 365 or 395 nm afforded the product in 15 min, with a 72–73% of Zr radioactivity being associated with the trastuzumab [92]. The radioimmunoconjugate was evaluated in athymic nude mice bearing a subcutaneous SKOV-3 tumor, showing a specific accumulation of radioactivity in the tumor. 

The preparation of the ^89^Zr radiocomplex for molecular imaging (Scheme 3E) is an interesting example of tetrazole photoconjugation chemistry [93]. By irradiating the ligand and the antibody (trastuzumab) at 365 or 395 nm for 10 min at room temperature, the immunoconjugate was obtained, with an average of 1.5 accessible DFO ligands per mAb. However, the nature of the conjugate bonds formed is uncertain. The complex was radiolabelled with ^89^Zr at pH 8.5. The obtained radiotracer is stable for 24 h at 37 °C in serum and 3 days in sterile PBS. As usual, the radiocomplex was tested towards athymic nude mice bearing SKOV-3 xenografts exhibiting specific tumor uptake [93].

^67^Gallium is a radioisotope (t_1/2_ = 3.26 days) that emits intense Auger electrons and γ-rays (8 to 887 keV) and was the first radiometal developed for radioimmunoscintigraphy and Immuno-Single Photon Emission Computed Tomography (Immuno-SPECT) [82]. The oxidation state +3 dominates gallium coordination chemistry, with a preference for hard ligands, especially hexadentate ones. ^111^Indium is a radioisotope that decays (t_1/2_ = 2.80 days) with Auger electron emissions (2.72 keV) that is useful for SPECT imaging and targeted RIT [82]. In the corresponding antibody conjugates, DTPA chelating ligand (DPA = diethylenetriaminepentaacetic acid) was used for the stabilization of the metal center. Owing to its higher ionic radius compared to gallium, the indium center can accommodate additional ligands.

With these valuable pieces of information in hand, Holland’s group reported the synthesis of gallium and indium complexes exploiting the photoactivable aryl azide group bound to a diethylenetriaminepentaacetic acid (DTPA) moiety [94]. It is worth noting that DTPA is well known in medicinal chemistry, especially in Single Photon Emission Computed Tomography (SPECT) applications. For example, ^111^In-satumomab pendetide is studied for the diagnosis of ovarian and colorectal carcinomas [95].

**Scheme 4:**
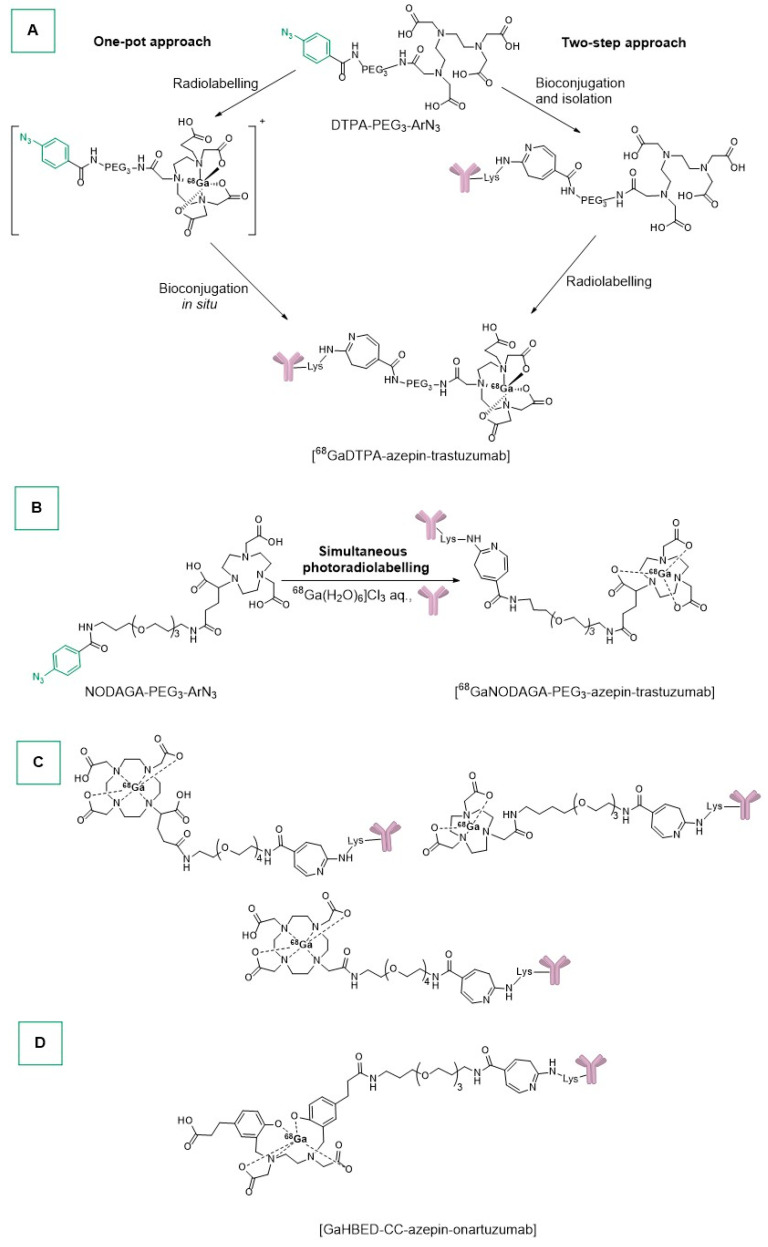
(**A**): Synthesis of trastuzumab complexes via one-pot and two-step approaches. (**B**): Simultaneous photo-radiolabelling of NODAGA-PEG_3_-ArN_3_. (**C**,**D**): Gallium radioimmunoconjugates reported by Holland.

^68^Ga and ^111^In complexes were obtained with two different approaches by reacting the DTPA-based ligand and [^68^Ga(H_2_O)_6_]Cl_3_ (aq.)/[^111^InCl_3_] (aq.) in an acetate buffer (pH 4.4) at room temperature (Scheme 4A). The one-pot procedure consisted of dissolving the ligand (DTPA-PEG_3_-ArN_3_) in water and adjusting the pH to 4–4.5 using an acetate buffer, followed by addition of ^68^GaCl_3_ and ^111^InCl_3_. The pH was then adjusted to 7.7–8.3 using Na_2_CO_3_. The reaction mixtures were exposed to UV light and purified with SEC and centrifugal filtration, with final radiochemical conversion between 3.9 and 5.4% (SEC analysis) and radiochemical purities higher than 80% [94]. The two-step approach required the spin filtration pre-purification of the antibody (trastuzumab). The antibody was then photoconjugated to the ligand (pH range 7.9–8.3), followed by PD-10-SEC purification eluting with PBS, and then radiolabelled with ^68^Ga^3+^ (radiochemical conversion of 10.0–12.8%). 

The same group reported the synthesis of a gallium complex, starting from the ligand and the formulated monoclonal antibody trastuzumab (Herceptin^TM^, which contains histidine, α,α-trehalose dehydrate, and polysorbate 20); the process is completed in 20 min, including synthesis, conjugation, and purification (Scheme 4B) [96]. The ligand was pre-radiolabelled with ^68^Ga, pre-buffered to pH 8.0, mixed with pre-purified trastuzumab, and irradiated with UV light, affording [^68^GaNODAGA-azepin-trastuzumab] in sterile PBS. The method was also tested without pre-purification of the antibody and using the formulated Herceptin^TM^. In vivo tests on athymic nude mice bearing a subcutaneous SKOV-3 tumor showed that the complex remained in circulation for more than 6 h post-injection with a specific tumor uptake (confirmed by competitive binding studies using a blocking dose of non-radiolabelled trastuzumab). 

Gallium-pegylated radioimmunocomplexes were reported by the same group [36]. In particular, the pegylated ligands (NOTA, DOTA, and DOTAGA) were prepared and pre-radiolabeled with [^68^Ga(H_2_O)_6_]^3+^ (Scheme 4C). Afterwards, the reaction mixtures were irradiated for 15 min at room temperature in the presence of an aliquot of pre-purified trastuzumab. The three radioimmunoconjugates were successfully obtained with radiochemical yields between 11 and 18% (measured with PD-10-SEC or SEC-UHPLC). Other gallium immunoconjugate complexes were synthesized incubating the antibody onartuzumab with a 5-fold excess of ligand at room temperature and at pH 8–9 (Scheme 4D) [97]. The stability of complex [^68^GaHBED-CC-azepin-onartuzumab] was evaluated by incubation in human serum albumin at 37 °C, showing a loss of small amount of radioactivity (<5%) after 3 h. Finally, MKN-45 or PC-3 xenografted mice were treated with the radioimmunoconjugate. In the MKN-45 model, tumors were easily visualized, and the radiotracer remained in circulation for 6 h. Moreover, [^68^GaHBED-CC-azepin-onartuzumab] targets very well the c-MET in vivo only in the MKN-45 model, showing that the PC-3 model has an insufficient c-MET concentration for optimal visualization in PET imaging.

## 3. Maleimide, Succinimide, and Other Linkers for Metal-Based ADCs 

**Scheme 5:**
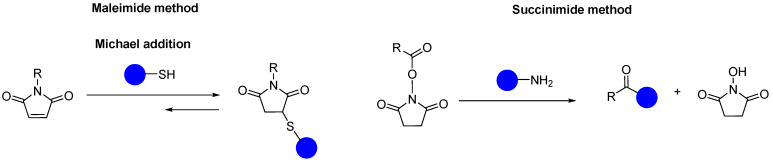
Maleimide and succinimide conjugation methods.

The conjugation promoted by the use of Michael acceptors such as maleimide (e.g., maleimide thiol reaction) is still in use, despite the fact that it usually does not reach full conversion and is also perturbed by cross reactivity, as, for example, in the case of antibody Lys-NH_2_ residues at high pHs (Scheme 5) [98]. In addition, thiosuccinimide adducts can undergo retro-Michael addition or thiol exchange reactions under physiological conditions. 

On the other hand, NHS-esters [99] are useful in amide bond formation to prepare highly functionalized compounds with peptides [100,101], natural products [102], and other bioactive compounds [103]. 

In this chapter, examples of metal-based ADCs prepared with maleimide and succinimide conjugation methods are discussed in detail.

**Scheme 6:**
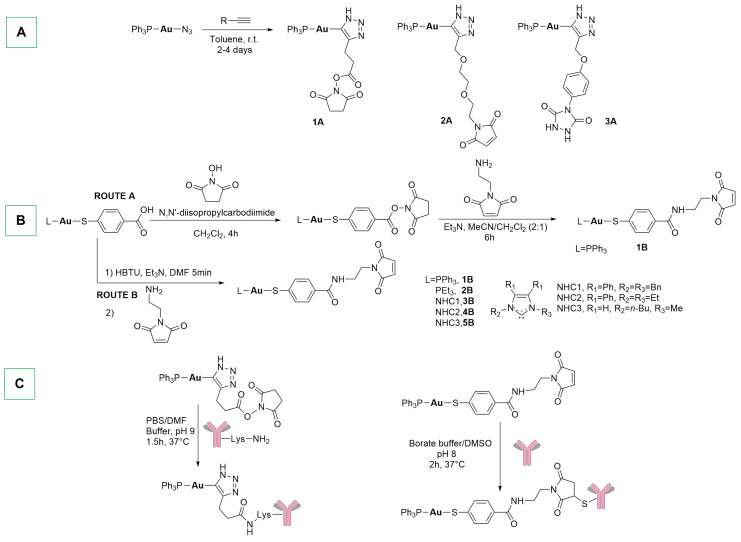
(**A**,**B**): Synthesis of linker ligands for gold complexes, as reported by Lewis and Contel. (**C**): Bioconjugation via succinimide and maleimide of gold complexes, as reported by Lewis and Contel.

In 2019, the Lewis and Contel groups reported for the first time the synthesis of antibody gold-based conjugates (AGC) using two different approaches [104]. Specifically, the first synthetic strategy involved a copper-free cycloaddition to link the gold fragment [N_3_-Au-PPh_3_] to the alkyne-derived linker (Scheme 6A). The second synthetic option includes the formation of an amide bond between a linker with a terminal amine and a gold thiolate complex containing a free carboxylate group (Scheme 6B, route A). All complexes were obtained with moderate yields (30–60%) and exhibited excellent stability (up to 40 days) in DMSO or DMSO:PBS. Compound **1A** was conjugated using the *N*-hydroxy succinimide ester fragment, which can react with antibody-available lysine residues (Scheme 6C). It should be remembered that this method could generate a heterogeneous ADC population due to the high number of lysines accessible in the antibody. On the contrary, compound **1B** can easily react with antibody cysteine residues through the maleimide moiety, after the reduction of the antibody interchain disulfide bond. The two trastuzumab conjugates were obtained with 55% (lysine) and 70% (cysteine) yields and were characterized with MALDI-TOF and size-exclusion HPLC, showing a drug-to-antibody ratio (DAR) of 2.7–3.2 for lysine conjugation and 2.7 for cysteine conjugation, in addition to stability for over a week in human serum. The conjugated products were tested on different cancer cell lines (MCF-7, BT-474, and MCF-10A) and displayed enhanced cytotoxicity in HER2-positive cells (MCF-7 and BT-474), with the cysteine conjugate significantly more cytotoxic than the starting complex. All of the tested compounds were less cytotoxic on non-cancerous MCF-10A cells, as compared to the cancerous ones. 

Also, the cysteine method can yield conjugates with heterogeneous DARs; for this reason, engineered antibodies with added cysteine residues that provide thiols for conjugation have been produced (e.g., Thiomab, the engineered trastuzumab) [105]. A few years later, the Contel group modified the synthetic protocol for the preparation of complex **1B**, obtaining different derivatives with reduced reaction times and high yields (Scheme 6B, route B) [106]. The antibody Thiomab was reduced with the decoupling agent (2-carboxyethyl)phosphine (TCEP) in order to free the blocked cysteine residues, as confirmed by the Ellman test. In doing so, the native cysteine residues are reduced and therefore need be reoxidized by dehydroascorbic acid, which binds the interchain cysteines while leaving the new cysteine reduced. After that, the complexes were conjugated to Thiomab through maleimide chemistry. Interestingly, under these conditions, only the phosphine complexes were able to conjugate the antibody, whereas the same reaction did not proceed in the case of Au-NHC (NHC = *N*-heterocyclic carbene) complexes. The gold-PR_3_ immunoconjugates were obtained with high purity (>95%), exhibited high binding affinity to HER2 receptor, and were stable up to 7 days in human serum. These ADCs were tested against different HER2-positive (MCF-7, BT-474, and SKBR-3) and HER2-negative (MDA-MB-231, and MCF-10A) cell lines, and demonstrated the targeting efficacy of site-specific gold-containing ADCs against HER2-positive cancer cells. 

**Scheme 7:**
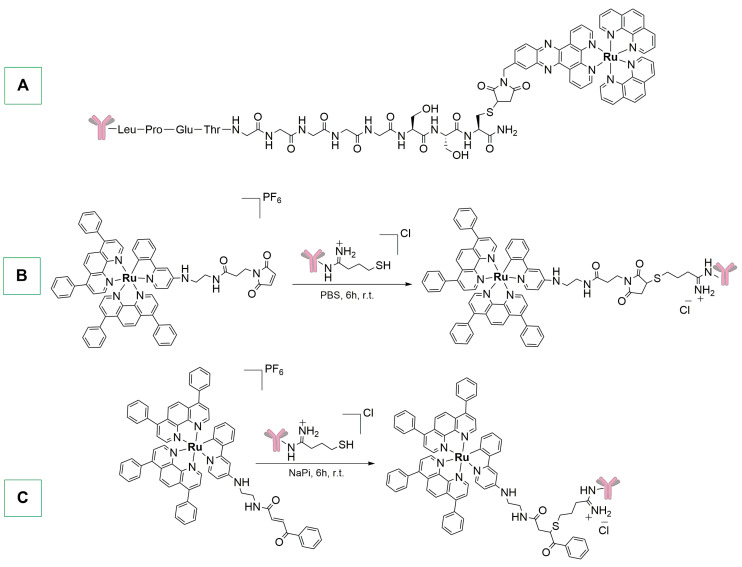
(**A**–**C**) Synthesis of Ru polypyridyl immunocomplexes.

In 2020, Gasser and colleagues reported the synthesis of the first ruthenium polypyridyl nanobody conjugate through maleimide conjugation chemistry (Scheme 7A) [107]. The complex contains three main building blocks: (i) the [Ru(phen)_2_(dppbz)]^2+^ fragment; (ii) the 7C12 nanobody (NB), known for specifically binding EGFR expressing cells; and (iii) a peptide chain with a poly-glycine unit. The conjugation between the engineered nanobody and the ruthenium complex by the enzyme sortase A was performed at 30 °C for 4 h with a molar ratio of 1:1:10 (SrtA:NB:complex). MALDI-TOF MS analysis showed a homogeneous population of a single-conjugated NB with a molecular weight of 17.7 kDa [107]. This Ru-NB derivative was tested towards A431 human epithelial cancer cells (epidermoid carcinoma), and confocal imaging showed co-localization of Ru-NB with epidermal growth factor receptor (EGFR). Receptor specific uptake was evaluated on A431 (EGFR-positive) and MDA-MB-435S (EGFR-negative) cell lines, showing a high amount of ruthenium in the EGFR-overexpressing cells. Nevertheless, irradiation of Ru-NB at 480 nm did not cause any cytotoxic effect on A431 cells or production of reactive oxygen species (ROS).

Similar complexes were conjugated to cetuximab via maleimide and benzoyl acrylate conjugation methods, as reported in Scheme 7B,C [108]. The antibody was pre-treated with Traut’s reagent (2-iminothiolane) to thiolate the amino groups of accessible lysines. The complexes were then conjugated in the presence of PBS or NaPi buffers. Unfortunately, neither immunoconjugated product induced statistically relevant reduction of cell viability on SQ20B cancer cells.

To learn more, detailed reviews on ruthenium polypyridyl bioconjugated complexes were reported by Martínez-Alonso/Gasser [109] and Correira/Morais [110].

**Scheme 8:**
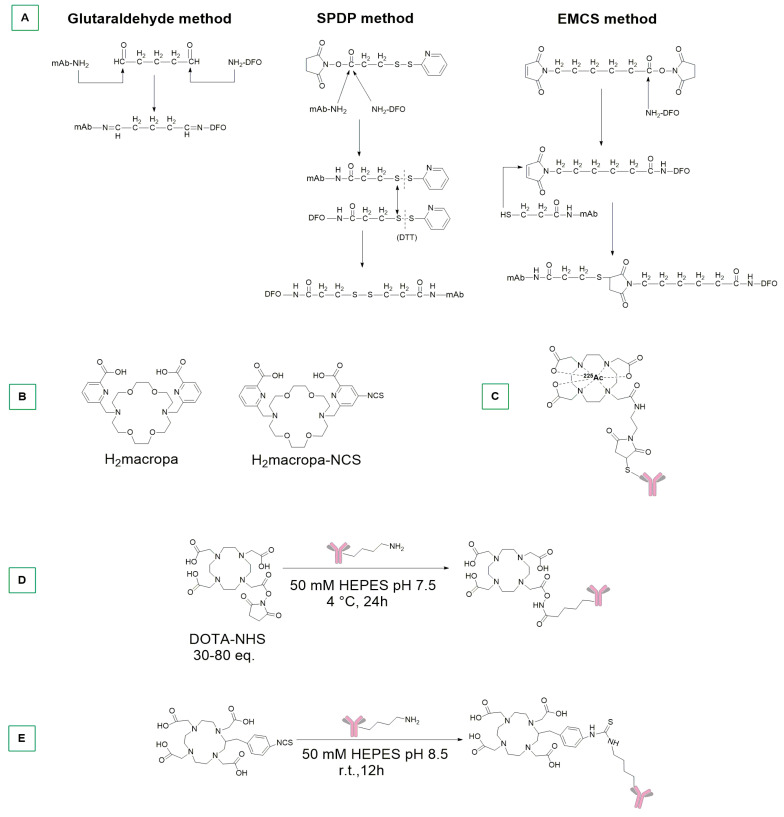
(**A**): Different methods used for antibody conjugation. (**B**): Examples of macropa ligands. (**C**): SS DOTA-MMA-mAb-^225^Ac, as reported by Abergel. (**D**,**E**): Labelling method reported by Scheinberg for the three-arm and four-arm constructs.

In 1988, the Koizumi group reported the synthesis of ^67^Ga-labeled antibodies exploiting glutaraldehyde, pyridyl disulfide, and maleimide conjugation methods (Scheme 8A) [111]. With glutaraldehyde, DFO was conjugated with mAb through Schiff’s base formation. On the contrary, the pyridyl sulfide method consists of using succinimidyl 3-(2-pyridyldithio)propionate (SPDP) as key reagent. The synthetic procedure involves the following: (i) the introduction of 2-pyridyl disulfide into both mAb and DFO exploiting SPDP; (ii) reduction of the 2-pyridyl disulfide residue of mAb by dithiothreitol; and (iii) conjugation of a thiol group to mAb and 2-pyridyl disulfide of DFO by thiol-disulfide exchange reaction. Regarding the maleimide method, a thioether bond was formed between the amino groups of mAb and DFO using SPDP and *N*-ε-malemidocaproyl-oxysuccinimide ester (EMCS) (Scheme 8A). 

The conjugated antibodies obtained using these three different approaches were then radiolabelled by incubating DFO-mAb and ^67^GaCl_3_ for 30 min at room temperature, with high radiochemical yields. Specifically, the radiocomplex obtained with glutaraldehyde method was detected in human osteogenic sarcoma KT005-xenografted mice at high concentrations (24 h and 48 h post-injection), with a high accumulation in the liver.

^225^Ac is a radioactive isotope that decays to ^209^Bi with a 10-day half-life, producing, in total, 4 α and 3 β^−^ emissions [82]. In the literature, there are some reported examples of Ac complexes with DTPA, DOTA, and macropa chelators [112,113] (Scheme 8B) as well as representations of their in vivo stability [114]. 

In this context, Abergel and colleagues developed an ^225^Ac ADC for the treatment of small-cell lung cancer through maleimide chemistry [115]. Antibodies were pre-incubated for 5 min at 45 °C and the radionuclide (0.05 M HCl solution) was added in large excess (200-fold) for 2 h at 45 °C (Scheme 8C). The conjugated product was subsequently tested on 293T cancer cells with and without DLL3 (Delta-Like 3 Protein) expression. IC_50_ values were found to be dependent only on radioactivity and completely independent of the antibody concentration. The conjugates were also tested in patient-derived xenografts (PDX) and, when a solid tumor reached 100 mm^3^, animals were injected intraperitoneally with a pre-dose of SS huIgG1 (nonspecific antibody), SS DOTA-MMA-huIgG1 (negative control), SS DOTA-MMA-N149-^225^Ac and SS DOTA-MMA-SC16-^225^Ac (N149 and SC16 are site specific antibodies), SS DOTA-MMA-N149-PDB, and SS DOTA-MMA-SC16-PDB (positive control, PDB = pyrrolobenzodiazepine). Both actinium-based ADCs exhibited similar efficacy, with life expectancy extended from 36 to 64 days. Notably, an analogous study was also conducted with ^117^Lutetium [115].

In 2013, Kurihara et al. prepared a ^64^Cu-DOTA-Trastuzumab conjugate and then tested it as probe to determine the presence of primary or metastatic HER2-positive breast cancer via PET imaging [116]. The radioimmunoconjugate was synthesized by premixing the prepurified trastuzumab IgG (Herceptin^TM^) and DOTA mono *N*-hydroxysuccinimide ester in water. Then, after purification with a PD-10 column, DOTA-Trastuzumab in PBS was exchanged to sodium acetate buffer by filtration. Finally, the conjugate was radiolabelled by adding ^64^CuCl_2_ to an acetate buffer solution for 1 h at 40 °C. The radioimmunoconjugate was tested in six patients: three primary cancers in stage IIA-IIB and three metastatic cancers with brain and hilar node metastases. The results confirmed successful tumor uptake and, compared with other radiometals, the detection time was lower (e.g., ^89^Zr five days after injection) [116]. 

Finally, a year later, Scheinberg and colleagues described a labelling method to produce stable mAb (rituximab and HuM195) conjugates with ^225^Ac [117]. In particular, the conjugation between the ligand three-arm-DOTA (Scheme 8D) and the antibody was conducted at 4 °C for 24 h in a HEPES buffer (pH = 7.5). Similarly, the conjugation with the ligand four-arm-DOTA (Scheme 8E) was performed at room temperature for 12 h. Afterwards, the metalation was performed at 37 °C for 2 h and the radioconjugates were injected into healthy BALB/c mice, showing that the four-arm derivative exhibited a higher accumulation than the three-arm one. In addition, the radioimmunoconjugates were evaluated against mice containing Set2-Luc cells, showing that the 1.11 kBq dose was able to cause reduction of tumor burden between days 14 and 26. 

## 4. Metal-Based ADCs with Direct Metal–Antibody Conjugation and Stochastic Conjugations with Radiometals

In the field of metal-based ADCs, some researchers have developed conjugation methods based on the direct reaction between the metal center and a functional group of the antibody of interest.

**Scheme 9:**
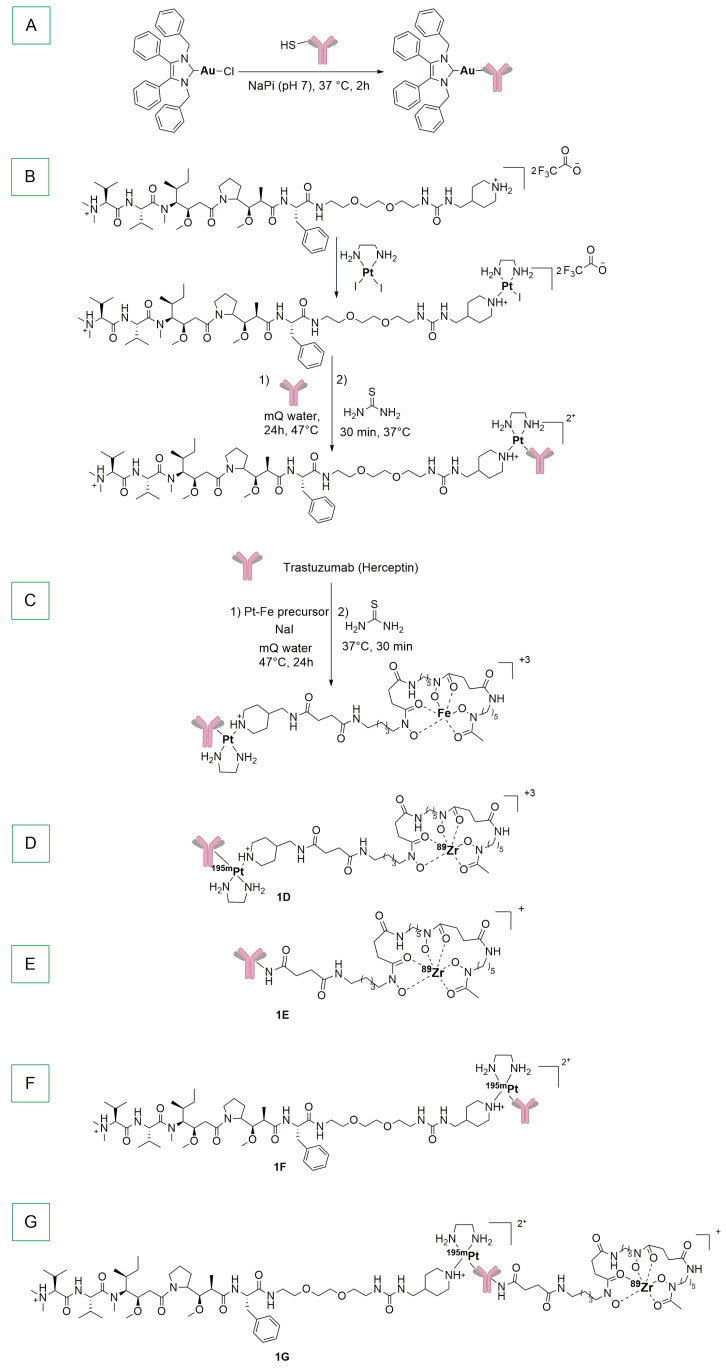
(**A**): Direct sulfur-mAb conjugation. (**B**–**G**): Immuno complexes synthesized by Merkul and co-workers.

In this context, the Bernardes group reported the synthesis of a gold complex directly bound to the engineered trastuzumab (Thiomab) via a Gold-S bond (Scheme 9A) [118]. Using five equiv. of NHC-Au-Cl per light chain for 2 h at 37 °C, the thio-gold adduct was synthesized, taking advantage of a single cysteine residue per light chain and the absence of modification in the heavy chain of the antibody. The antibody retained the capacity to bind the HER2 receptor of the SKBR3 breast cancer cells, and furthermore promote a moderate enhancement in the antiproliferative activity, compared to NHC-Au-Cl.

In an interesting work, Merkul and colleagues described the synthesis of a Pt-ADC using trastuzumab as mAb and auristatin F as cytotoxic payload [119]. The group developed an heterogeneous “stochastic conjugation” which avails of histidine residues of native unmodified antibodies for stable drug-linker attachments [120]. Their technology works in two steps. The first step consists of the coordination of a *N*-heterocyclic ligand to platinum(II), which, being charged, improves the solubility of the final complex. The second step is the stochastic conjugation of the Pt-payload to an unmodified mAb or a site-specific conjugation to cysteine residues in mAb fragments. The payload was transformed by adding a linker that ends with a piperidine moiety and that acts as complexing agent by substituting the iodide ligand of the [Pt(en)I_2_] precursor. The conjugation step was performed in a multigram scale (16 g) with high purity (Scheme 9B). Trastuzumab was conjugated by reacting the complex in mQ water at 47 °C for 24 h, followed by addition of thiourea for 30 min at 37 °C (quenching step) to ensure stability and reproducibility of the conjugates. The role of the thiourea is to remove the thermodynamically labile Met-bound antibody, leaving only the thermodynamically stabile His-bound antibody [121]. The platinum immunocomplex was administered in non-tumor-bearing mice, and showed no relevant weight loss (4% with 30 mg/kg, 10% with 60 mg/kg), thus indicating that high dosages of this ADC are tolerated. The work also proved that eight among the nine mice, in which NCI-N87 and JIMT-1 (Kadcyla resistant line) cells were implanted, survived when treated with this immunoconjugate [120]. 

The same group extended this technology to other metal centers such as iron or radioactive zirconium, obtaining Fe-Pt and Zr-Pt heterobimetallic complexes with 2.5 DAR with high yield (Scheme 9C).

The zirconium heterobimetallic complex was obtained by reaction of the Fe-Pt complex with a ^89^Zr precursor. The radiozirconium conjugate was tested on HER2-positive NCI-N87-bearing nude mice, and showed a good stability of the immunoconjugate [120]. Similar homo- and hetero-bimetallic immunocomplexes were prepared and tested in nude mice bearing human epidermal growth factor receptor 2-positive gastric cancer xenograft line NCI-N87 [122]. In particular, compound **1D** (Scheme 9D) showed similar levels of ^195m^Pt and ^89^Zr in blood, tumors, and most of the healthy tissues. Conversely, in sternum and femur a higher uptake of ^89^Zr was detected. No differences were found among complexes, the only exception being **1F**, which was associated with a higher liver uptake. Compound **1G** (Scheme 9G) showed fast blood clearance and liver uptake through PET imaging. This study highlighted that the metallic fragment [Pt(en)]^+^ remained an inert component of the ADC after in vivo administration, unlike cisplatin.

The analogues of **1D** and **1F** with nonradioactive Pt were tested in HER2-overexpressing cell lines SKOV-3, BT-474, NCI-N87, and SK-BR-3, showing IC_50_ in the range of 20–200 pM also in the trastuzumab and ado-trastuzumab emtansine-resistant JIMT-1 cell line [123]. Notably, the toxicity of **1F** was higher than that of the non-immunoconjugate complex. Biodistribution studies in NCI-N87-bearing mice revealed similar radiozirconium levels in the tumors and all organs for both conjugates, as well as a good stability in vivo. Other platinum-derived ADCs have been described by Contel and del Solar in a review of five years ago [60].

**Scheme 10:**
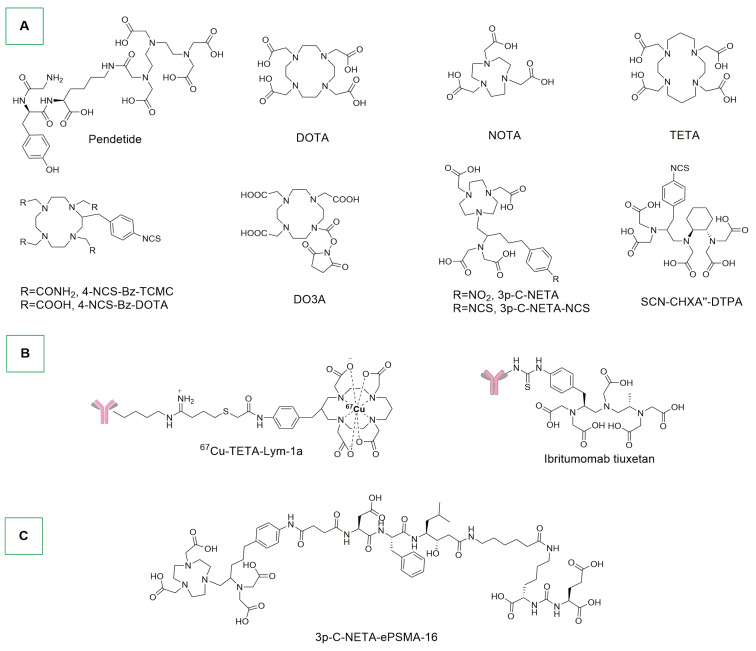
(**A**): Chelating ligands useful for radiometal conjugation. (**B**): Radiocopper conjugate and ibritumomab tiuxetan. (**C**): 3p-C-NETA-ePSMA-16 ligand.

Indium-labelled antibodies have been reported since the late 1980s [124]. For example, Ryan et al. labelled with ^111^InCl_3_ (efficiency: 80–100%) human IgM mAb produced by fusing a mouse myeloma cell line to lymph node lymphocytes of different patients affected by breast adenocarcinoma. The radioimmunoconjugate was mixed in a sterile saline solution and administered in different patients while observing, by scintigraphy, its presence in metastatic breast lesions, as well as in liver and spleen [124]. ^111^In capromab pendetide (Scheme 10A) was intensively studied in the early 2000s for the detection of prostate cancers [125]. This study includes 7E11-C5.3, a murine monoclonal antibody conjugated to a chelating linker: glycyl-tyrosyl-(*N*-diethylenetriaminepentaacetic acid)-lysine hydrochloric acid. The antibody recognizes an intracellular epitope that reacts with more than 95% of prostate adenocarcinomas when analyzed in vitro with immunohystochemistry [126]. For the detection of lymph node metastasis, ^111^In capromab pendetide demonstrated positive predictive values of 72%. In more than 80,000 patients who were exposed to the antibody conjugate, no serious allergic reactions were observed, and in clinical trials, adverse effects were detected in only 4% of the 529 patients. Unfortunately, low sensitivity for viable tumor lesions was observed for clinical diagnosis of prostate cancer and the ^111^In capromab pendetide was not useful for the identification of cancer lesions in bones. However, despite these limitations, it is still available in the market, although other indium-based antibody–drug conjugates were studied, such as ^111^In-satumomab pendetide (for colorectal and ovarian carcinomas) [95]. 

Radioactive copper is a metal used in medicinal chemistry coupled and conjugated to monoclonal antibodies. In aqueous conditions, copper chemistry is dominated by the Cu^2+^ cation, which is, as well known, Jahn–Teller distorted. To exclude in vivo demetallation of Cu-mAb conjugate and the consequent accumulation of the radioactivity in background tissues such as liver, spleen, and kidneys, aza-macrocyclic-based chelating ligands like DOTA, NOTA, and TETA are used (Scheme 10A) [82]. Philpott and co-workers developed a murine mAb that binds to a lipid antigen enriched in human colon carcinoma cells, and which presents excellent immunospecificity for colorectal adenocarcinoma [127]. After a pre-incubation between BAT and the antibody (mAb 1A3) at 37 °C for 30 min, followed by gel-filtration chromatography purification, radiolabelling of the conjugated ligand (benzyl-TETA-mAb 1A3) with ^64^Cu was performed at room temperature for 30 min at pH 5.5. Finally, gel-filtration purification led to the final conjugate. The radiometal-mAb conjugate exhibited greater specificity in the detection of human colorectal tumors than did [^18^F]-FDG (known for the detection of false positives) and no adverse effects were observed [82,127].

In 1995, De Nardo and colleagues reported the preparation of a ^67^Cu-TETA-Lym-1a. Lym-1-IgG2a is a murine anti-lymphoma mAb that reacts with a membrane antigen found on malignant cells of patients with B-cell lymphoma. The conjugation was obtained by exploiting the selective formation of a bond between the NH_2_ terminus of the light chain of Lym-1 and the ligand coordinated to the metal (Scheme 9B) [128]. The process was carried out in 0.1 M tetramethyl ammonium phosphate (pH 8.8–9.1) for 30 min at 37 °C. Subsequently, the radiolabelling process was performed by incubating a 0.1 M HCl solution of radiometal ^67^Cu and ligand for 30 min at room temperature, followed by EDTA chelation of the non-specifically bound metals and final purification by centrifuged column filtration. The radioconjugate was then tested against non-Hodgkin’s lymphoma (NHL) in 12 human patients, leading to promising results. In fact, the radioconjugate efficiently targeted NHL in all of the patients, considering that 90% of them presented intermediate or high-grade stage III or IV chemotherapy-resistant lymphoma [129]. In another study, regression of the tumor (18% to 75%) was observed in patients after several days of infusion with the radioconjugate [130]. 

Other examples of ^64^Cu-DOTA-trastuzumab confirmed the ability of the radiocomplex to bind HER2 receptors of both HER2-positive and HER2-negative in metastatic breast cancer patients [131]. The observation that some HER2-negative patients showed higher uptake than some HER2-positive patients aligns with the finding that certain HER2-negative patients benefit from trastuzumab treatment. Moreover, the radioconjugate was also evaluated towards metastatic brain tumors; the results indicated its ability to pass through the blood–brain barrier [132]. 

Chen and co-workers synthesized in a similar way ^64^Cu-Cetuximab and ^90^Y-cetuximab [133]. The copper conjugate was tested in PET in human head and neck squamous-cell cancer (HNSCC, UM-SCC-22B, and SCC1) mouse models. Only UM-SCC-22B showed higher tumor accumulation of the tracer, although EGFR expression was lower than that of SCC1. On the other hand, only the SCC1 tumor responded well to cetuximab therapy. In fact, the UM-SCC-22B tumor proliferation rate increased after the treatment, compared to the control group. Using ^90^Y-cetuximab, UM-SCC-22B tumor growth was inhibited and four of the seven treated tumors disappeared. The authors hypothesized that the regression of the tumor was due to radiation accumulation and not to antibody delivery [133]. In addition, other potentially suitable ^64^Cu and ^67^Cu radioimmunoconjugates for PET imaging were reported by Holland and colleagues [134]. 

^90^Yttrium is a radiometal that decays through β^−^ emission and has a half-life of 2.67 days [82]. The energy of its emission is useful for the treatment of poorly vascularized tumors. Frequently, DOTA and CHX-A″-DTPA ligands (Scheme 10A) are the chelating agents used for its stabilization, but in view of the slow complexation kinetics at room temperature of DOTA and the decreased stability in vitro of DTPA derivatives, other ligands have been developed (e.g., 3p-C-NETA, Scheme 10A) and used with high radiochemical yields under mild conditions [82]. 

In this context, Witzig and colleagues reported in 2002 the first phase III study to evaluate the efficacy and safety of ^90^Y ibritumomab tiuxetan (Scheme 10B) [135]. In particular, ibritumomab is a murine IgG_1_ anti-CD20 antibody. The study pointed out that the immunotherapy with ^90^Y increased the percentage of partial or complete response from 56 to 80% with excellent response rates also found in chemoresistant patients affected by B-cell NHL [136]. 

The effect of ^90^Y-SCN-CHXA″-DTPA-6D2 mAb (ligand in Scheme 10A) (anti-melanin recombinant antibody) was evaluated by Thompson and colleagues in A2058 melanoma tumor-bearing mice [82,137]. After a premixing process between the protein solution and CHX-A″ DTPA ligand in conjugation buffer at room temperature for 18 h, the radiometal (MCl_3_) was incubated for 1h at 37 °C. After that, a solution of EDTA was used to remove any free radiometal. The same procedure was used also for ^166^Ho. This holmium conjugate showed better results than ^90^Y, reducing tumor growth in A2058 melanoma tumor-bearing mice, with a measurable therapeutic effect that was comparable to rhenium conjugates already known in the literature [138]. 

Other examples of Yttrium mAb conjugates were reported by Lindén and co-workers using ^90^Y-DOTA-epratuzumab [139]. The study highlighted that this yttrium mAb conjugate can be used and administered in a multiple-week infusion with good results against non-Hodgkin’s lymphoma. The authors noticed a correlation between the therapeutical response and the CD22 (a sugar-binding transmembrane protein) expression. 

Technetium is known for its wide range of oxidation states, from negatives (−1) to highly positive (+7) and it was widely used to develop SPECT radiotracers [82]. During the 1990s, different ^99m^Tc-mAbs received approval, but after few years they were withdrawn. This was also the case for ^99m^Tc-nofetumomab merpentan, which was used for imaging in lung, gastrointestinal, breast, ovary, and pancreas cancers. Other examples include ^99m^Tc-arcitumomab for targeting the carcinoembryonic antigen on colorectal cancers, ^99m^Tc-sulesomab for imaging infections and inflammations in patients with suspected osteomyelitis, and ^99m^Tc-votumumab for the detection of CD15 expression in colorectal tumors [82]. 

Radioactive lutetium immunocomplexes have been reported in the literature as ^177^Lu-3p-C-NETA-NCS-trastuzumab conjugates [140]. The conjugation was obtained by adding an excess of ligand (3p-C-NETA-NCS, Scheme 10A) to a solution containing trastuzumab and gently stirring the resulting mixture for 16 h at room temperature [141]. Afterwards, the antibody–ligand solution was mixed with a solution of LuCl_3_ in HCl 0.05 M and incubated at room temperature or 37 °C. The radiolabelling was extremely rapid, taking the complexation only one minute. The bioconjugate was stable in human serum for 2 weeks, and the study on ZR-75-1 bearing nude mice proved that the highest tumor uptake was obtained after 72 h post-injection, with low radioactivity in liver and kidneys. A similar ligand, 3p-C-NETA-ePSMA-16, which contains a PSMA targeting vector (Scheme 10C), was also evaluated (PSMA is a highly restricted prostate epithelial cell integral membrane glycoprotein expressed in most of prostate carcinomas [142]). Heating the ligand at 90 °C for 5 min with a solution of ^177^LuCl_3_, it was possible to obtain high radiochemical yields, and the resulting conjugate had proved stable in PBS and mouse serum up to 24 h post-incubation [142]. 

^212^Pb can be produced from ^232^U and is a β emitter with a half-time of 10.6 h. 

Martin Brechbiel and colleagues reported in the early 2000s the synthesis of a Pb-4-NCS-Bz-TCMC/4-NCS-Bz-DOTA-mAb conjugate [143]. The antibody was premixed with the ligand. After the dialysis of the mAb, an aqueous solution of 4-NCS-Bz-TCMC or 4-NCS-Bz-DOTA (Scheme 10A) was added to the antibody solution, and the mixture was kept at room temperature for 18 h. Subsequently, a solution of ligand in ammonium acetate buffer was added to a solution of ^203^Pb in 0.1 HCl and incubated for 30 min at 37 °C. The purified conjugates were incubated with 2 mL human serum for 168 h at 4 °C. This product was stable for 138 h at 4 °C and 24 h at 37 °C [143]. A ^212^Pb-TCMC-Trastuzumab conjugate was evaluated in ovarian cancer patients after they had failed multiple prior therapies (carboplatin in combination with taxane/gemcitabine) [144]. The radioconjugate was administered in a single intraperitoneal injection of 7.4 MBq/m^2^. The maximum blood concentration (6 nCi/mL) occurred after 18 h and there was no evidence of localization of the radioconjugate in normal organs, despite prolonged retention within the peritoneal cavity. ^212^Pb-TCMC-Trastuzumab did not cause late toxicity, after 1 year post-treatment, up to 27.4 MBq/m^2^ [145].

^213^Bismuth is a radioelement that decays by α emission followed by two β^−^ or β^−^-α-β^−^ emission to ^209^Bi. Its lifetime is very short (45.6 min) and in spite of that, in the literature there are some examples of Bi mAb conjugates. For example, Scheinberg and colleagues reported the synthesis of ^213^Bi-SCN-CHXA″-DTPA-J519 mAb conjugate (ligand in Scheme 10A) [146]. J519 antibody targets the external domain of PSMA. After an elution of Bi (HNO_3_ 1.0 M solution) in an anion exchange resin with 0.1 HCl/NaI, the antibody–ligand solution (7–10 mg/mL) was added, and the reaction proceeded at 22 °C at pH 5 for 10–20 min, after which it was quenched by adding a solution of EDTA [146,147]. The conjugate was tested against LNCaP spheroids (LNCaP cells are androgen-sensitive human prostate adenocarcinoma), demonstrating a 1-week delay in growth with respect to untreated spheroids. The in vivo model was also investigated with LNCap-xenografted mice (tumor xenografted in mice of 8 weeks of age), resulting in an increase in the survival rate (54 days on ^213^Bi-SCN-CHXA″-DTPA-J519) compared to the untreated group (31 days). Another study conducted by Sgouros and colleagues [148] concerned the treatment of patients with relapsed acute myeloid leukemia with ^213^Bi-HuM195 by intravenous injection for 5 min. HuM195 is a humanized version of mouse antibody M195, a monoclonal antibody that reacts with CD33 antigen on myeloid and monocytic leukemia cells. The authors found that the concentration of ADC in red marrow, liver, and spleen increased rapidly. In fact, leukemia is characterized by an irregular proliferation of hematopoietic cells in bone marrow. Therefore, proliferation of leukemia cells to extramedullary sites such as liver and spleen is common because of the high vascularization of those organs.

Tsuji’s group evaluated the labelling of OTSA101 antibody to different actinium chelated complexes [149], showing that between DOTA, DOTAGA, and DO3A (Scheme 10A) derivatives, the maximum labelling efficiency was obtained with DOTAGA. To obtain the conjugation between the ligand and the antibody, a solution of OTSAS101 was reacted with a solution of DOTA, DOTAGA, or DO3A in a borate buffer for 16 h at 37 °C. The target conjugates were obtained using ^225^AcNO_3_ dissolved in HCl, in the presence of tetramethylammonium acetate and ascorbic acid. The chelate-conjugated OTSAS101s were added to the actinium solution and the mixture was incubated at 37 °C. The stability of the labelled complexes was evaluated in murine serum at 37 °C, with more than 96% of ^225^Ac that remained bound to the adduct for 7 days at 37 °C. The maximum tumor uptake was obtained with ^225^Ac-DOTA-OTSA101 (30.7 ± 16.5%), with absorbed doses in lungs, kidneys and intestine significantly higher than those of the other conjugates. In addition, ^255^Ac-DOTAGA-OTSA101 showed the highest tumor-to-bone marrow ratio, indicating that the most promising chelating agent is DOTAGA. This ligand allows ^255^Ac-labelled OTSA101 with high binding affinity and radiochemical yields to be produced, the use of which results in doses being highly absorbed to the tumor and limited amounts of doses being absorbed to bone marrow. The evaluation of the antitumor effects in tumor-bearing mice showed that all three mAb conjugates exhibit strong tumor shrinkage, with a mixture of necrotic and tumor cells identified four days after treatment with a lower number of proliferating tumor cells than the untreated tumor. 

Flavell’s group reported the synthesis of a ^225^Ac mAb conjugate for prostate cancer treatment [150]. The antibody, YS5, targets CD46, an antigen that is homogeneously expressed in prostate cancer [150]. The buffer of antibody YS5 was exchanged from HEPES to NaHCO_3_ and then was diluted in a carbonate/bicarbonate buffer, and 20 equiv. of *p*-SCN-Bn-DOTA in DMSO was added to the solution containing 5 mg of YS5 antibody, and incubated at 37 °C for 1 h. The reaction mixture was purified with a PD10 gel-filtration column. Radiolabelling was performed by mixing [^225^Ac(NO_3_)_3_] with HCl solution, NH_4_OAc, L-Ascorbic acid, and YS5-DOTA at 40 °C for 2 h, followed by purification processes. The number of DOTA chelators on YS5 was determined with the MALDI-TOF technique, which revealed 8.7 ± 0.2 equiv. of DOTA conjugated to each YS5, with a final yield of 39.4 ± 4.4%. After 14 days, 55% of the radiolabelled conjugate was intact in human serum. The conjugate was evaluated on two different prostate cancer cell lines (22Rv1 and DU145), revealing that, for 22Rv1, most of the ^225^AcDOTA-YS5 was internalized in the first 30 min of incubation, whereas in the case of DU145 cells, the internalization of the conjugate is slower (from hours to one day). Mice bearing a 22Rv1 tumor were treated with 0.5 μCi of the conjugate via the tail-vein. Tumor uptake increased from 24 h (11.64% ± 1.37%) to 96 h (28.58% ± 10.88%) and 408 h (37.78% ± 5.89%). The uptake was higher in the tumor than in nontargeted organs for all timepoints except 24 h, when kidneys were particularly affected. The γ-H2AX test revealed that after 7 and 14 days, the number of DNA double-strand breaks increased with the dose. Moreover, xenografted mice were treated with 0.25 or 0.5 μCi (microcuries) of ^225^AcDOTA-YS5, showing significant inhibition of tumor growth in an activity-dependent manner, while mice in the control group showed rapid tumor growth. 

Jurcic and coworkers developed ^225^Ac-DOTA-SCN-Lintuzumab and tested this ADC against acute myeloid leukemia [151]. This derivative was prepared by the labelling of ^225^Ac to DOTA-SCN in an acetate buffer at 55–60 °C for 30 min. Then, after mixing it with lintuzumab in a carbonate buffer for 30 min at 37 °C, the final product was obtained with 10% yield after the purification process. Although remission was not observed, bone marrow blasts were reduced in 67% of the tested patients [151]. 

**Scheme 11:**
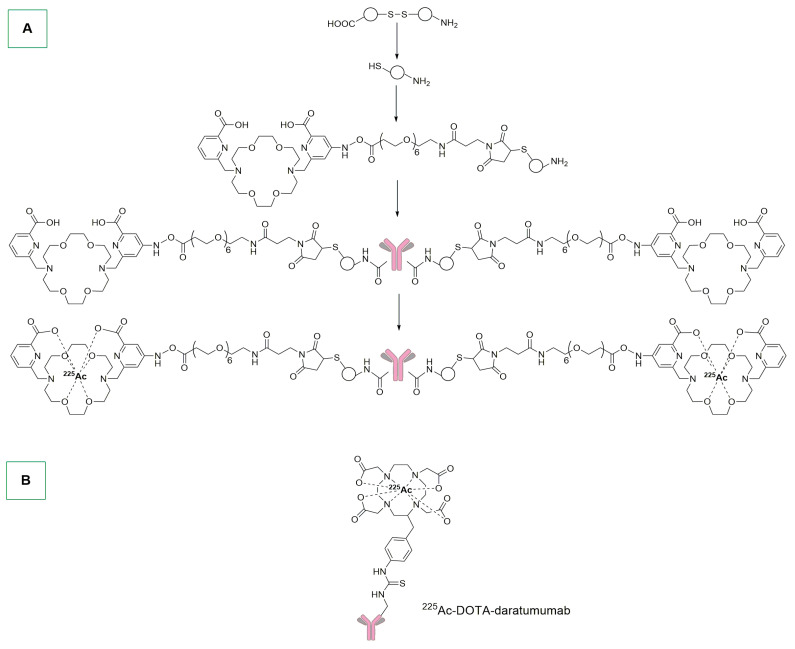
(**A**): SpyTag-SpyCatcher conjugation. (**B**): Actinium radiocomplex, as reported by Dawicki.

In 2020, Geyger, Fonge, and colleagues developed an actinium-SpyTag-SpyCatcher-Macropa-Nimotuzumab (Scheme 11A) [152]. The SpyTag/SpyCatcher system is used for irreversible conjugation of recombinant proteins. The SpyTag peptide reacts with the SpyCatcher protein (12.3 kDa) to form an intermolecular isopeptide bond [153]. The metal labelling was performed by reacting actinium nitrate in 0.05 M HCl and nimotuzumab-SpyTag-ΔN-SpyCatcher-macropa in ammonium acetate 150 mM at 37 °C for 50 min. The in vivo efficacy was evaluated in MDA-MB-468 xenograft with two doses of 450 nCi (nanocuries) administered via tail-vein at day 0 and day 14. Notably, the mice did not show weight loss, and in the treated group, 6/8 mice showed positive responses. All mice were euthanized when the tumor exceeded 2000 mm^3^, and the control group reached that volume value after 29 days. In the treated group, mice were euthanized after 56 (2 mice), 64 (2 mice), and 75 (1 mice) days, and one mouse survived past 120 days. 

Dawicki and colleagues reported the study of ^225^Ac-daratumumab conjugate against multiple-myeloma tumors (Scheme 11B) [154]. The conjugation of DOTA to the antibody did not compromise the CD38 binding on Daudi cells, and the radiolabelling with ^225^Ac permitted the inhibition of the proliferation of these cells in vitro. The conjugate was also tested in Daudi and KSM28BM-xenografted mice exhibiting antitumor effects, reducing tumor growth. Furthermore, the drug was considered safe for mice, without affecting distinctly their weight or blood parameters.

## 5. ADCs Bearing Natural or Nature-Inspired Organic Payloads

Many natural or nature-inspired organic molecules are known to exhibit interesting anticancer properties. However, most of them display poor selectivity towards cancer cells as well as stability or solubility issues. One of the main strategies used to overcome these problems is the development of ADCs bearing these organic molecules, in order to deliver the cytotoxic payload directly to the tumor site [67,155,156,157,158,159,160]. In this section, the most important examples reported in the last five years concerning the use of natural or nature-inspired molecules as payloads in ADCs with anticancer properties are discussed.

In this broad context, one interesting biologically active molecule is dolastatin 10, which was isolated for the first time in 1980s from Dolabella auricularia (a mollusk) [161]. This organic compound shows a good antiproliferative activity towards different types of cancer [162]. Its anticancer properties were attributed to its interaction with tubulin, a key protein involved in the formation of microtubules, and which is essential for cell division [67]. 

**Scheme 12:**
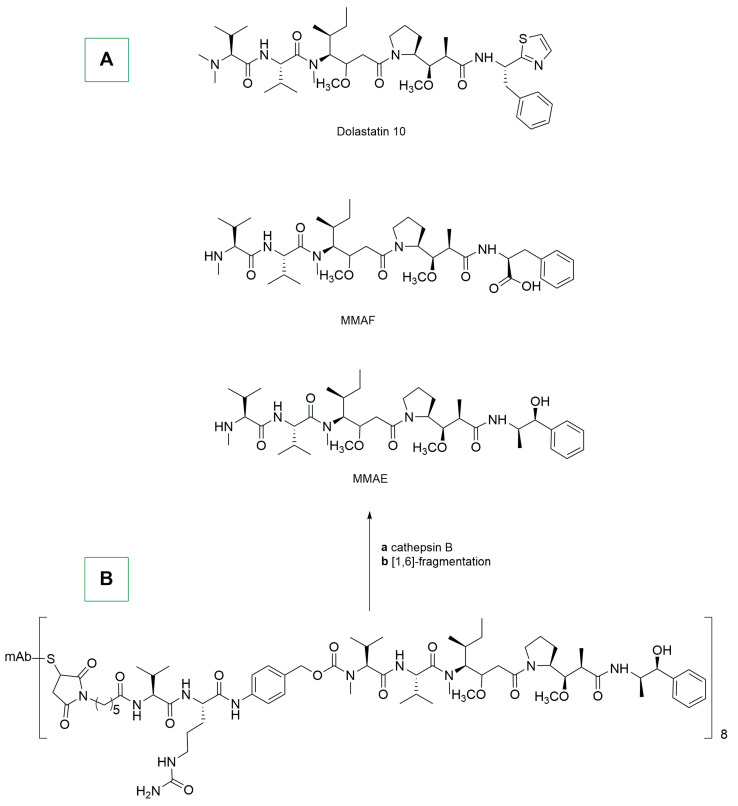
(**A**): Dolastatin 10, MMAF, and MMAE. (**B**): Brentuximab vedotin and its possible cleavages in the biological environment.

With the aim of enabling the conjugation of dolastatin 10 with antibodies, two different dolastatin 10 derivatives, namely, MMAF and MMAE, were recently synthesized (Scheme 12A). More in detail, MMAE is a sequence of four aminoacids (monomethylvaline (MeVal), valine (Val), dolaisoluine (Dil), and dolaproine (Dap)) ending with a carboxy-terminal amine norephedrine moiety. Conversely, MMAF presents a phenylalanine instead of norephedrine [163,164]. 

The conjugation between dolastatin 10 derivatives with a chimeric monoclonal antibody (cAC10) was particularly efficient with MMAE, as demonstrated by the Senter and Zhu groups [165,166]. It should be remembered that cAC10 is a monoclonal antibody specifically directed against cluster of differentiation 30 (CD30), a cell membrane protein frequently overexpressed in Hodgkin lymphoma and systemic anaplastic large-cell lymphoma (sALCL) [67]. The conjugation process between MMAE and cAC10 was performed using the maleimide method. Specifically, protease-cleavable dipeptide linkers were added to the *N*-terminal position of MMAE through a self immolative *p*-aminobenzylcarbamate spacer. The final ADC, known as brentuximab (Adcetris), was obtained in high yields (>80%) and contained eight attached drugs per mAb (Scheme 12B) [67,166]. In Scheme 12B is also reported a possible degradation pattern leading to free MMAE in the biological environment.

Brentuximab was administered as a single 1.8 mg/kg intravenous infusion over 30 min on day 1 of a three-week cycle, up to a maximum of 16 cycles [165]. The tumor objective response rate (ORR) for the modified intention-to-treat population (mITT) was 69%. Overall, responses were reported for 27 patients, which included 11 patients with complete response (CR) and 16 patients with partial response (PR). Sixteen patients attained a response within 2 months after the first dose, seven patients had responses throughout the treatment, and five patients received a subsequent autologous stem cell transplant (ASCT). 

Most of the patients showed reductions in the sizes of their target lesions. Regarding the safety of the treatment, 100% of patients reported one or more treatment-emergent adverse events (TEAE) of any grade and 97% of patients reported one or more drug-related TEAE of any grade; the most common were hematologic toxicity, serum chemistry abnormalities, and neuropathy. 

Polatuzumab vedotin-piiq (Polivy) is an ADC that specifically targets CD79b antigen, which is mainly present in B-cell malignancies. In this ADC, the mAb is conjugated to MMAE in a way similar to that of brentuximab vedotin [67,167]. Polivy received approval in 2019 by FDA for its utilization in combination with rituximab and bendamustine [168]. 

In the same year, a study regarding polatuzumab Vedotin was conducted in patients with transplantation-ineligible relapsed/refractory (R/R) diffuse large B-cell lymphoma (DLBCL) [169]. All patients received bendamustine (90 mg/m^2^) intravenously on days 2 and 3 of cycle 1, and then days 1 and 2 of subsequent cycles, and rituxumab (375 mg/m^2^ on day 1 of each cycle) or obinutuzumab (1 g on days 1, 8, and 15 of cycle 1, and day 1 of subsequent cycles). The group also treated with polatuzumab vedotin received 1.8 mg/kg of this ADC on day 2 of cycle 1 and on day 1 of subsequent cycles. Patients were treated for up to six 21-day cycles [169]. Treatment with polatuzumab vedotin combined with bendamustine-rituximab resulted in higher complete response rates and reduced the risk of death by 58% compared with bendamustine-rituximab [169]. 

Another MMAE conjugate is enfortumab vedotin (Padcev), which was approved by FDA in 2019, and in which MMAE is conjugated to an mAb that specifically targets Nectin-4, releasing MMAE within the cells [67,170]. A phase III study was conducted in patients with advanced urothelial carcinoma, dividing the 608 patients into two groups: 301 patients received enfortumab vedotin and 307 classical chemotherapy. The incidence of treatment-related adverse events was similar in the two groups (93.9% and 91.8%, respectively). This ADC prolonged survival, as compared to standard chemotherapy, in patients who had previously received platinum-based treatments and PD-1/PD-L1 inhibitors (checkpoint inhibitors that block the activity of PD-1/PD-L1 immune checkpoint proteins) [171]. 

Tisotumab vedotin-tftv (Tivdak) has recently emerged as a promising ADC for the treatment of solid tumors such as metastatic cervical cancer [67]. This compound was approved by FDA in 2021 for adult patients and includes an MMAE moiety conjugated to tisotumab, an mAb that targets the tissue factor (TF). In 2020, a phase I/II study on cervical cancer patients (55 people) was reported [172]. Specifically, 51% of the patients had received more than two prior lines of treatment and 67% had prior bevacizumab and chemotherapy treatment. The most common treatment-emergent events were anemia, fatigue, and vomiting. The median duration of response was 4.2 months, and four patients responded longer than 8 months. The authors reported that the safety profile was consistent with other MMAE-based ADCs except for epistaxis and conjunctivitis (in fact, TF is highly expressed in the nasal epithelium). 

In another study, 102 patients were enrolled and 101 of them received at least one dose of tisotumab vedotin, some obtaining a complete (7%) and partial responses (17%). The related adverse events comprised neutropenia (3%), fatigue (2%), ulcerative keratitis (2%), and peripheral neuropathies (2%). A serious treatment-related adverse event occurred in 13% of patients withsensorimotor neuropathy and pyrexia. In total, one death due to septic shock was considered to have been related to the therapy and three deaths were considered not to have been related to the treatment [173]. 

Belantamab mafodotin-blmf (Blenrep) is an ADC developed for multiple myeloma and approved by FDA in 2020. It consists of a monoclonal antibody that targets B-cell maturation antigen (BCMA), conjugated to MMAF [67]. A study on the efficacy of this ADC was reported in 2022, comprising patients with more than one line of treatment, and lenalidomide (LEN) and proteasome inhibitor (PI) exposure, as well as refractoriness to the last line of treatment. The study considered the combination of the ADC and pomalidomide (POM) and dexamethasone (DEX). POM was administered at 4 mg/day for 21/28 days, DEX 40 mg weekly in combination with belantamab mafodotin administered as a single dose of 1.92 mg/kg, 2.5 mg/kg, or 3.4 mg/kg on days 1 and 8. The most frequently reported adverse events were keratopathy, decreased best-corrected visual acuity, fatigue, thrombocytopenia, blurred vision, neutropenia, myeloma progression, lung infection, and myelodysplastic syndrome. Despite this, belantamab mafodotin has been considered a possible drug for multiple-myeloma treatment [174]. 

Another dolastatin 10-derived ADC is disitamab vedotin, which was developed for the treatment of breast cancer and urothelial carcinoma. The mAb used targets HER2 receptor (epidermal growth factor receptor 2) and is conjugated to MMAE [175]. Different preclinical and clinical studies were reported on this ADC. In two recent phase II clinical trials on 107 patients with HER2-positive locally advanced or metastatic urothelial cancer demonstrated a promising efficacy with a manageable safety profile [176,177,178,179]. 

**Scheme 13:**
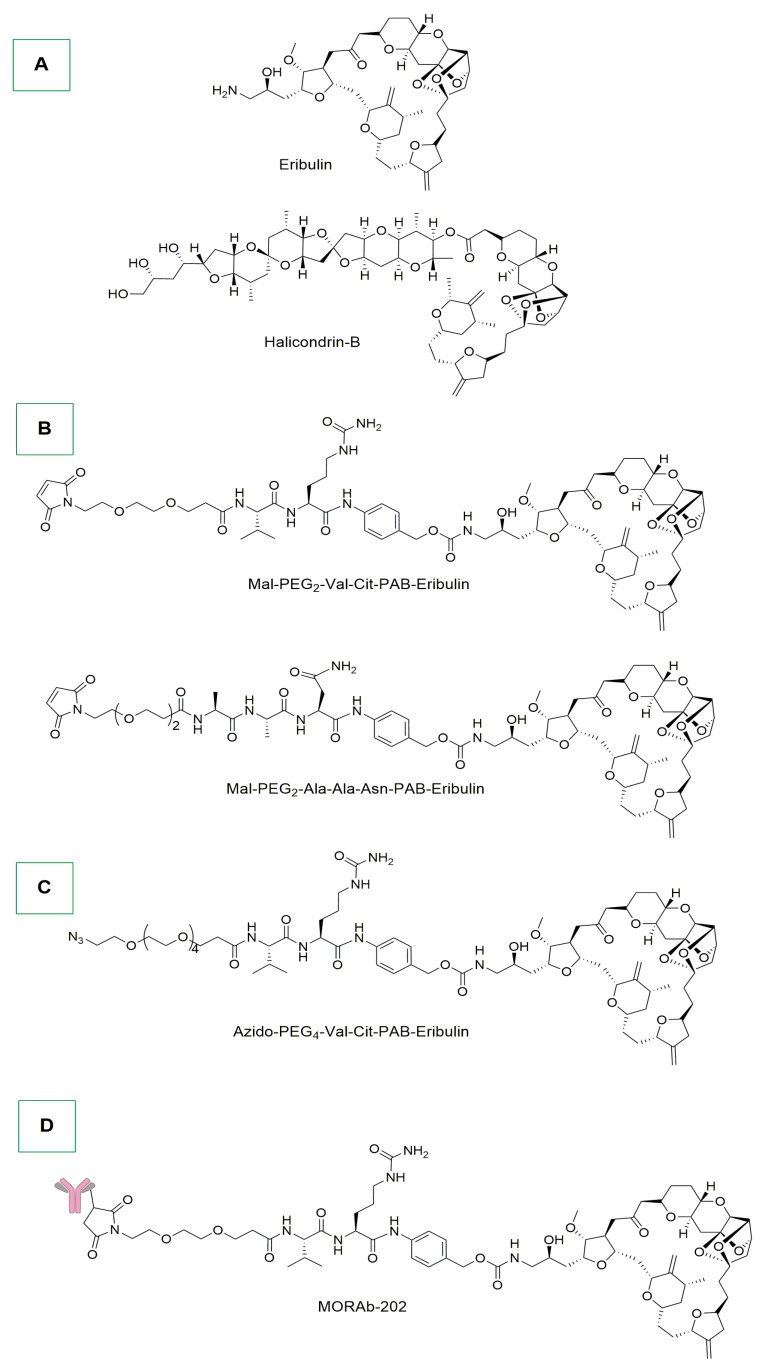
(**A**): Eribulin and halicondrin-B. (**B**): Maleimide linker eribulin. (**C**): Azide linker eribulin. (**D**): MORAb-202.

A second class of interesting ADCs bearing nature-inspired payloads is that of halicondrin B and eribulin derivatives. Halicondrin B, which was isolated for the first time from the marine sponge Halichondria okadai [180], is a natural product with anticancer properties, whereas eribulin mesylate (Halaven) is a synthetic analogue used as anticancer agent (Scheme 13A) [181]. The mechanism of action of halicondrin B and eribulin involves microtubule dynamics, that is crucial for cell division, affecting both the growth and shortening phases of microtubules [67].

In this context, Albone and colleagues reported in 2018 the preparation of eribulin ADCs through two different methods [182]. In the first approach, maleimido-linker-eribulin was conjugated to partially reduced antibody using a 1:6 (mAb:eribulin) molar ratio, 50% propylene glycol in DPBS for 4 h at room temperature (Scheme 13B).

In the second approach, which is based on the alkyne–azide click chemistry, *N*-hydroxysuccinimide-dibenzylcyclooctyne (DBCO) was added to 50% propylene glycol and mixed thoroughly. Afterwards, an equal volume of Ab was added at a molar ratio of 1:4 (Ab:DBCO) and left for 1 h at room temperature [182]. Finally, azido-linker-eribulin (Scheme 13C) was added, and the click-conjugation was performed overnight.

From a biological point of view, a similar ADC, namely MORAb-202, was tested on patients (older than twenty years old) with FRα-positive (Folate Receptor α)-expressing solid tumors, who had failed to respond to standard therapy (Scheme 13D). The ADC was administered intravenously at doses of 0.3 to 1.2 mg/kg once every three weeks. Leukopenia and neutropenia were observed in 45% of patients. The MTD (highest dose of a drug or treatment that does not cause unacceptable side effects) was not reached, which therefore suggests promising anticancer properties for MORAb-202 [183]. 

**Scheme 14:**
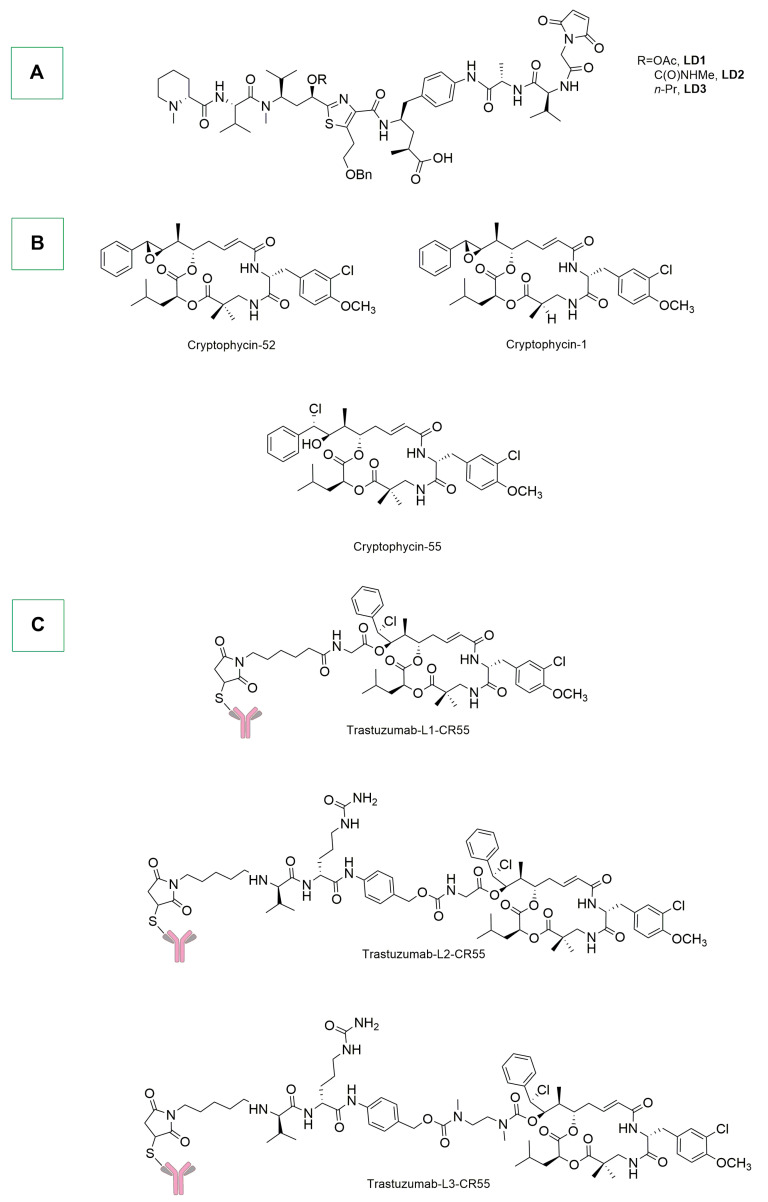
(**A**): Tubulysin linkers, as reported by Nicolaou. (**B**): Cryptophycin derivatives. (**C**): Cryptophycin-derived ADCs.

Tubulysin is another natural molecule that has anticancer properties due to its abilities of inhibiting microtubule formation and disrupting microtubule dynamics. This compound was isolated for the first time from myxobacteria *Archangium gephyra* and *Angiococcus disciformis* [184]. Some tubulysin derivatives have been incorporated in ADCs, targeting them to the tumor site and limiting their systemic toxicity. Different examples have been reported [185,186,187], and the most recent one comes from the Nicolaou group. In particular, the authors described the synthesis and biological evaluation of tubulysin-derived ADCs which were obtained in a site-specific manner, taking advantage of free cysteine residues of trastuzumab (Herceptin, Scheme 14A) [188]. The reduced mAb (thiol concentration between 1.8 and 2.2 mol per mol of antibody) in tris buffer was conjugated by adding 15% dimethylacetamide and 5 molar excess of linker drug, and then keeping at room temperature for 2 h. Three ADCs demonstrated target-specific in vitro cytotoxicity on SKBR3 and SKOV3 HER2-positive cell lines, and all exhibited excellent stability in cynomolgus primate plasma at 37 °C for over 7 days. In vivo evaluation of these ADCs was performed in an NCI-N87 mouse tumor model with a 3 mg/kg dose. Only herceptin-LD3 ADC showed a significant reduction of tumor volume [188].

Similar ADCs were obtained with cryptophycin, a natural molecule isolated for the first time from cyanobacteria (genus Nostoc) (Scheme 14B) [189]. It belongs to the class of depsipeptides (peptides in which one or more amide groups are replaced by the corresponding ester) and their primary mechanism of action involves the inhibition of microtubule assembly, which is essential for cell division [190]. In 2020, Yang and colleagues reported the synthesis of cryptophycin-55 ADCs in two steps [191]. The first step involved the reduction of interchain disulfide bonds of the antibody (trastuzumab) with TCEP. In the second step, maleimide conjugation chemistry was used for the conjugation between the maleimide-linker-CR55 and the modified antibody (Scheme 14C). The three obtained conjugates showed potent antiproliferative activity against HER2-positive SKOV3 and NCI-N87 cells, with IC_50_ values in the low nanomolar range. For HER2-negative or weakly-positive MDA-MB-231 cells the activity was significantly reduced. Their activity was also evaluated in SKOV-3 and NCI-N87 tumor-bearing nude mice; the results indicated that a treatment at 10 mg/kg and 5 mg/kg significantly delayed tumor growth, with compound T-L3-CR55 exhibiting the best activity. During the treatment, no significant changes in the mice’s body weight were observed.

**Scheme 15:**
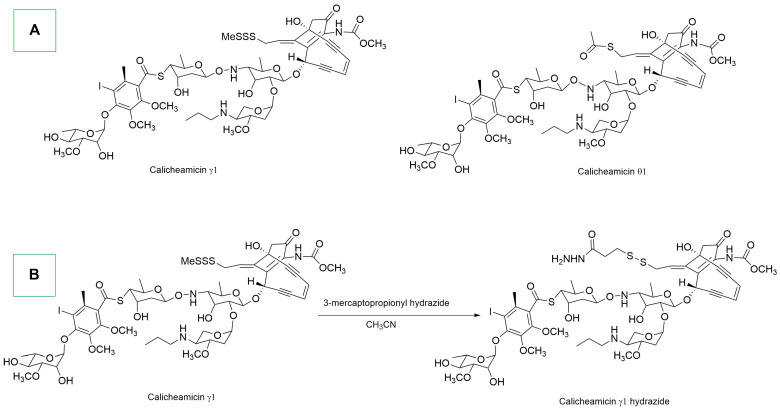
(**A**): Calicheamicin derivatives. (**B**): Calicheamin hydrazide linker.

Enediynes are a class of bicyclic natural compounds that can be isolated from various strains of actinobacteria (e.g., *Micromonospora* and *Streptomyces*) [192]. Their structure is composed of two acetylenic groups conjugated to a double bond, forming a 9- or 10-membered ring (Scheme 15A). Enediynes, especially Calicheamicin derivatives, can undergo Berman cyclization [193], generating diradical species that can abstract hydrogens from DNA’s deoxyribose sugar, leading to DNA strand break and cell death [67]. In the last decade, systematic meta analyses and in-depth studies on the topic have been reported [194,195,196,197,198].

In 2017, the ADC inotuzumab ozogamicin (Besponsa) was developed by Pfizer for the treatment of B-cell precursor acute lymphoblastic leukemia [199]. In this ADC, the monoclonal antibody is conjugated to a Calicheamicin hydrazide moiety. Specifically, Calicheamicin hydrazide derivatives were prepared by the reaction of calicheamicin γ1 and 3-mercaptopropionyl hydrazide in acetonitrile at 4 °C for 24 h (Scheme 15B). Subsequently, the mAb, at a concentration of 5 mg/mL in 50 mM sodium acetate buffer, was oxidized with sodium periodate. The oxidized antibody was finally reacted with a 30-fold molar excess of calicheamicin γ1 hydrazide in DMF [200]. In 2021, inotuzumab ozogamicin was subjected to the INO-VATE Trial, which was conducted in adults with relapsed/refractory B-cell precursor CD22-positive acute lymphoblastic leukemia [201]. The response rate was significantly higher with inotuzumab ozogamicin versus standard-of-care chemotherapy (SoC chemotherapy), with duration of remission (DoR), progression-free survival, and overall survival (OS) findings that were better for inotuzumab ozogamicin, compared to SoC chemotherapy, in patients with CD22 positivity ≥ 90%. 

**Scheme 16:**
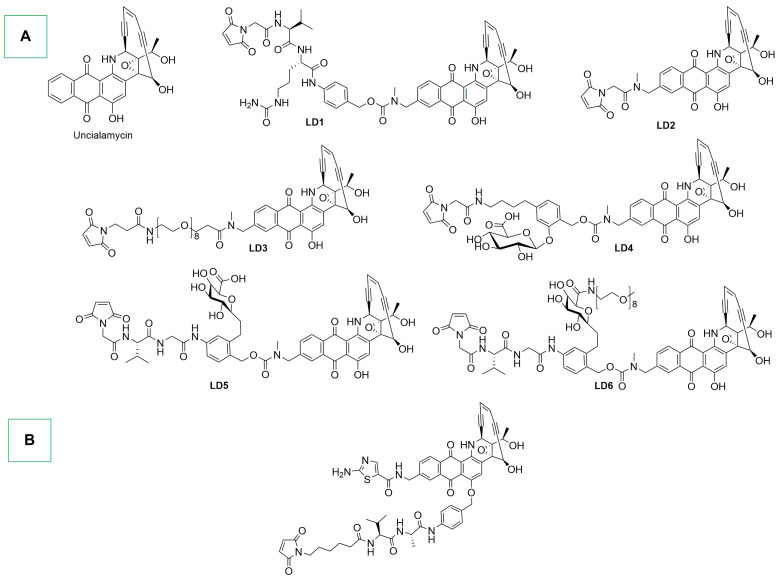
(**A**): Uncialamycin-derived linkers useful for mAb conjugation. (**B**): Uncialamycin derivative used by the Poudel group.

Within the broad class of enediynes, uncialamycin is an antibiotic which was isolated for the first time from the marine bacterium Streptomyces uncialis [67]. Uncialamycin-based ADCs were reported by Nicolaou and colleagues in 2021 [202]. The conjugation between the mAb and the linker-uncialamycin was performed by incubating a solution of reduced mAb (anti-T1Ab and anti-CD46Ab) in Tris buffer and in the presence of EDTA, with a 5 molar excess of linker-drug per mol of mAb (Scheme 16A). The mixture was vortexed and let sit for 90 min at room temperature. Then, 1.2 mol of N-acetylcysteine per mol of linker-drug was added, and the solution was vortexed and let sit for 20 min at room temperature [202]. The ADCs were tested in two patient-derived small-cell lung cancer xenograft models that had high levels of expression of both targets T1 and T2 (CD46). Notably, ADCs with non-cleavable linker-drugs (**LD2** and **LD3**) were found to be inactive. Conversely, in the case of cleavable linkers (**LD1** and **LD6**), antitumor activity was observed in a dose-dependent manner. In particular, **T1LD1** and **T2LD2** exhibited similar antitumor effects in LU95 and LU149, leading to complete tumor regression, which was sustained for more than 40 days. 

In 2020, Poudel and colleagues synthesized, with the maleimide–cysteine method, two similar derivatives bearing two tumor targeting antibodies: anti-CD70 and anti-mesothelin (Scheme 16B) [203]. H226 human lung carcinoma xenograft mice were treated with the two ADCs with a single dose at 0.1 μM uncialamicyn payload/kg dosed intraperitoneally. The study showed complete tumor growth inhibition for 6 weeks after the single dose. Other studies were conducted in vitro with similar derivatives [204]. 

**Scheme 17:**
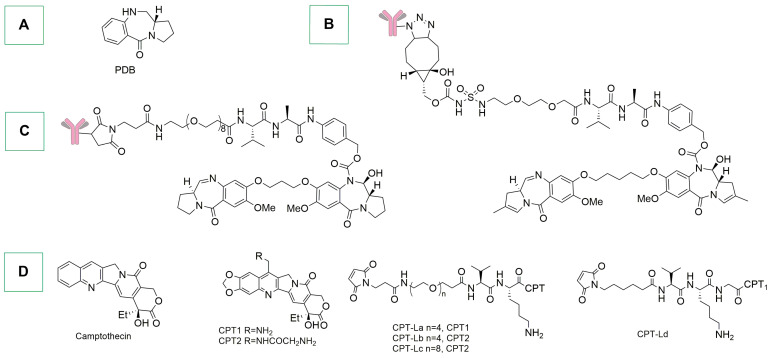
(**A**): Pyrrolobenzodiazepine. (**B**): PDB-ADC, as reported by Zammarchi. (**C**): PDB-ADC, as reported by Gregson. (**D**): Camptothecin and Camptothecin-derived linkers.

Pyrrolobenzodiazepines (PBDs) were discovered in cell cultures of *Streptomyces* in the 1960s [205]; the first isolated and characterized derivative was anthramycin (Scheme 17A) [206]. Since then, a number of naturally occurring PBDs have been isolated (e.g., sibiromycin and tomaymycin) [207,208] and investigated for their anticancer properties [67,209]. They act as alkylating agents, binding to the minor groove of DNA, as well as generating interstrand DNA crosslinks, interfering with DNA replication and transcription, leading to an apoptotic cell death [210,211,212,213,214,215]. 

In this context, Zammarchi and co-workers reported in 2022 the synthesis and application of an ADC containing a PDB payload (Scheme 17B) [216]. After an enzymatic trimming of the *N*-linked glycans in the Fc pocket of the antibody, the terminal GlcNAc was extended with 6-N_3_-GalNAc using GalNAc-transferase, allowing metal-free click conjugation of the payload [216]. The ADC was rapidly internalized by AXL-expressing tumor cells, releasing a PDB dimer that proved its DNA crosslinking properties. Moreover, in vivo tests on a variety of cancer xenograft models showed a markedly high antitumor activity. This promising anticancer activity was observed also in a monomethyl auristatin E-resistant lung-cancer model and synergized with PARP inhibitor olaparib in a BRCA1-mutated ovarian cancer model. The ADC showed also a good stability in vivo and was well tolerated [216]. 

In the same year, Gregson and colleagues reported the synthesis and biological evaluation of a PDB dimer-based ADC using the maleimide conjugation method (Scheme 17C) [217]. The obtained trastuzumab-containing ADC was evaluated in HER2-overexpressing NCI-N87 human gastric tumor xenografts, and, at a single dose of 3 mg/kg, a slightly delayed tumor growth was observed. On a JIMT-1 cell model (lower expression of HER2), the highest DAR of trastuzumab ADC resulted in delay of tumor growth at a single dose (10 mg/kg). Importantly, no mortality at doses of 25 to 30 mg/kg was observed in control ADC administration [217]. 

Camptothecin is a topoisomerases inhibitor discovered in 1966; it is isolated from bark and stem of *Camptotheca acuminata* (Scheme 17D) [218,219]. Some derivatives have been extremely well-studied as anticancer agents due to their ability of inhibiting topoisomerase I (TOPO-I), an enzyme important for DNA replication and transcription [67]. 

In this context, Jeffrey and colleagues described in 2020 a series of camptothecin-based ADCs using the maleimide thiol coupling (Scheme 17D) [67,220]. In particular, a PBS solution of fully reduced mAb (chimeric anti-CD30 antibody cAC10) was diluted 2-fold with propylene glycol and then a DMSO drug-linker solution was added over a 5 min period, resulting in drug-linker conjugation onto reduced interchain cysteine residues. The synthesized ADCs were first tested in vitro against CD30-positive cell lines. Specifically, the target cAC10 ADCs exhibited activity in the low ng/mL range on high CD30-expressing cell lines (L540cv, Karpas299, DEL and DEL BVR, and CPT-Lb and CPT-Lc). In particular, the ADC bearing the val-lys drug-linker CPT-LA showed activity only on DEL and DEL BVR cell lines and was inactive on L540cy and Karpas299. All three ADCs were inactive on L428 and DEL BVR. These ADCs were also tested in vivo on an L540cy-xenografted model; the results indicated that cAC10 CPT-Lb and cAC10 CPT-Lc cured the animals with a single 1 mg/kg dose. Unfortunately, a modest activity was performed by cAC10-GT ADC in both the single doses of 10 mg/kg and 30 mg/kg. Moreover, only cAC10-CPT-Lc was tested on the CD30 low L428 xenograft model, and both 1 mg/kg and 3 mg/kg doses resulted in tumor regression. 

A particular test using both antigen-negative and antigen-positive tumor cell xenografted mice (Karpas 299 and Karpas BVR cells implanted subcutaneously into SCID mice) was performed with a single dose of 3 mg/kg and 10 mg/kg of cAC10-CPT-Lc and cAC10-DT ADCs. This test proved that CPT-Lc ADC was more than 3-fold more active than cAC10-DT ADC.

**Scheme 18:**
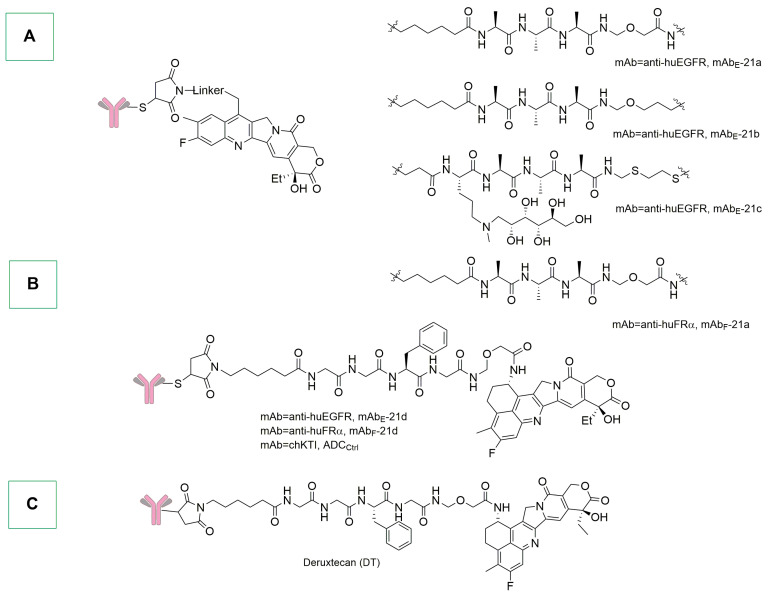
(**A**): Camptothecin-derived ADCs. (**B**): Other camptothecin-derived ADCs and control used in the experiments. (**C**): Trastuzumab deruxtecan.

A year earlier, Widdison and colleagues reported the synthesis and in vivo/in vitro evaluation of camptothecin-derived ADCs, taking advantage of the maleimide approach. The authors used humanized anti-huEGFR and anti-huFRα antibodies which bind human epidermal growth factor receptor 1 or human folate receptor-alpha (Scheme 18A,B) [221]. 

As expected, the synthesized ADCs were highly potent against antigen-positive cells and less potent towards nontargeted antigen-negative cells or antigen-positive cells in the presence of unconjugated antibodies that block the binding of the conjugate. Two ADCs (mAb_E_-21a and mAb_E_-21d) were evaluated in nude mice bearing human head and neck squamous cell carcinoma EGFR- + HSC-2 xenograft, as well as in nude mice bearing non-small-cell lung cancer (NSCLC) squamous cell H1703 xenograft (lower antigen expression). The ADCs showed similar dose-dependent antitumor activity in both models. The mAbE-21b was tested only in the H1703 model, showing a better activity than the other ADCs. Notably, all of the conjugates were tolerated in mice models.

Another ADC that contains a camptothecin-derived payload is trastuzumab deruxtecan (Scheme 18C) [222]. This drug has been extensively investigated in the recent years and has received approval by the FDA [223,224,225]. In 2022, a phase 3 trial involving patients with HER2-low metastatic breast cancer and who had received one or two previous cycles of chemotherapy was reported. In the HER2-+ cohort the median progression-free survival was 10.1 months, compared to the 5.4 months associated with classical chemotherapy treatments (i.e., eribulin, capecitabine, nab-paclitaxel, gemcitabine, and paclitaxel). Among all patients, the median progression-free survival was similar. The grade 3 events occurred in 52.6% of patients with trastuzumab deruxtecan and 67.4% of patients receiving classical chemotherapy.

**Scheme 19:**
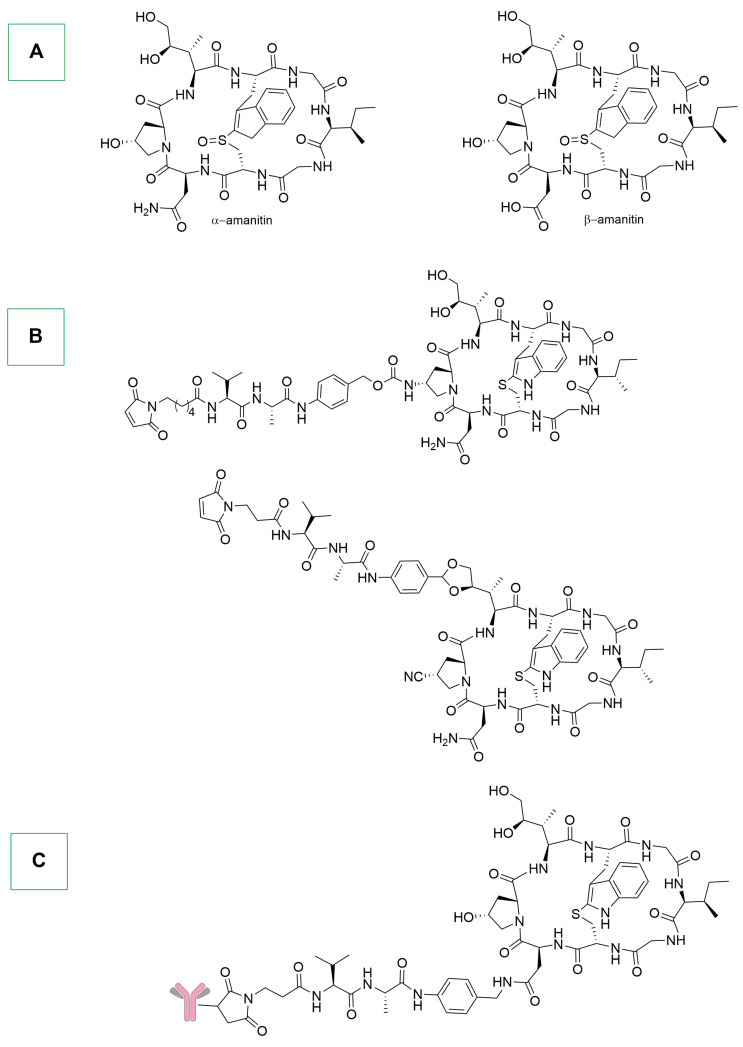
(**A**): Amanitin structures. (**B**): Amanitin derived linkers, as reported by Perrin. (**C**): Amanitin ADC, as reported by Raab.

Mushrooms frequently contain lethal toxins such as amatoxins, which, in this case, were extracted from Amanita phalloides [67,226]. Inhibition of RNA polymerase II, the key enzyme in the synthesis of mRNA in eukaryotic cells, is the principal mode of action of this type of toxins. The effect is particularly significant in rapidly proliferating cells such as those of the gastrointestinal tract and liver. The structure of two amatoxins, α-amanitin and β-amanitin, is reported in Scheme 19A [227]. 

In this context, Perrin and colleagues reported in 2021 the synthesis and conjugation of α-amanitin derivatives to anti-HER2 antibody T-D265C Thiomab (Scheme 19B) [228]. The Thiomab antibody was reduced with TCEP and, after dialysis, interchain disulfides were re-oxidized with dehydroascorbic acid. Afterwards, the two amanitin-linkers were site-specifically conjugated.

The ADCs were evaluated in vitro against three HER2-positive cell lines (SK-BR-3, SKOV-3, and JIMT-1) and HER2 negative ones (e.g., MDA-MB-231). Only in the case of SK-BR-3 cells, in which HER-2 is highly overexpressed, was the cytotoxicity significantly higher, demonstrating the therapeutic potential of amanitin derivatives for certain types of tumors.

On the other hand, Raab and co-workers developed an amanitin ADC for the treatment of proliferating and resting multiple-myeloma cells [229]. The amanitin derivative was conjugated to an anti-B-cell maturation antigen (BCMA) mAb (Scheme 19C) and was cytotoxic against both proliferating and resting myeloma cells in vitro, but not against BCMA-negative cells. Tumor regression at low doses was observed in subcutaneous and disseminated NCI-H929 murine xenograft models and was well tolerated in cynomolgus monkeys [230,231]. 

**Scheme 20:**
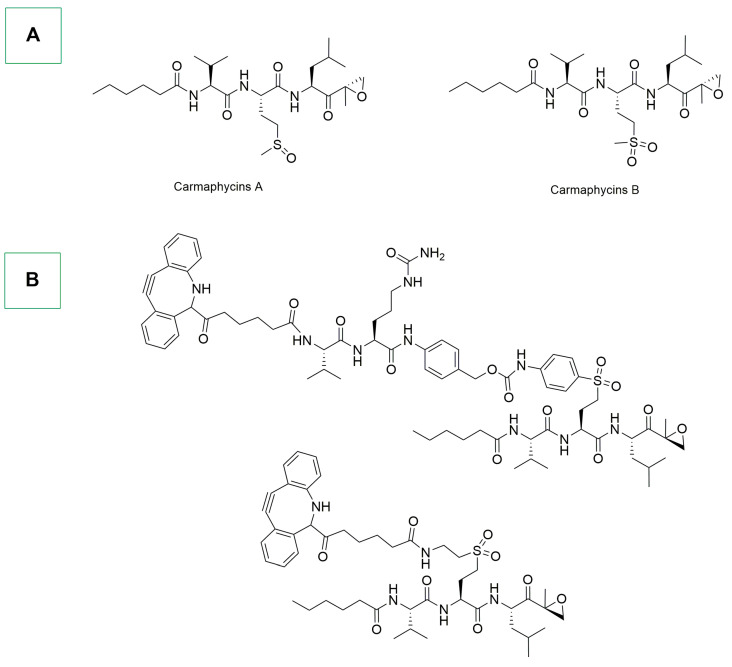
(**A**): Carmaphycin structures. (**B**): Carmaphycin-derived linkers, as reported by Gerwick.

The last class of molecules examined is that of carmaphycins, which are naturally occurring products isolated for the first time from cyanobacterium *Symploca* sp. and can act as proteasome inhibitors targeting the 20S proteasome in cancer cells (Scheme 20A). This pathway is responsible for degrading unwanted or damaged proteins within the cells. By its inhibition, carmaphycins disrupt cellular protein homeostasis, leading to apoptosis [67]. 

In 2019, Gerwick and colleagues reported the synthesis and conjugation of a library of carmaphycin B analogues with amine handles useful for conjugation on mAbs [232]. Based on the results on the NCI-H640 cell line and the ChT-L site of the proteasome, only two analogues were chosen for modification with a suitable linker (Scheme 20B). The conjugation was performed through alkyne–azide cycloaddition (SPAAC), with the alkyne part of the linker and an azide-containing antibody (trastuzumab). The obtained ADCs were tested on SKBR3 (HER2+) and MDA-MB-468 and MDA-MB-231 (HER2-) cell lines. Unfortunately, the ADCs did not show superior cell-killing against cancer cells, compared to the free antibody.

## 6. Conclusions

In this review, we have described the most important and recent examples of antibody–drug conjugates (ADCs) containing metal-based and nature-inspired payloads. Particular emphasis has been placed on the different synthetic strategies that can be employed to conjugate the antibody with the selected payload, highlighting the advantages and disadvantages of each method. Conventional methods, such as maleimide and succinimide conjugation methods, as well as classical condensation reactions, are the ones most commonly used for both metallodrugs and bioactive natural compounds payloads. However, alternative strategies, such as photoconjugation reactions, are rapidly evolving. These non-conventional methods allow for milder conditions and render the formation of target ADCs more selective, thereby limiting the side reactions and/or mixtures of ADCs, which are difficult to separate and identify. 

In addition to the different synthetic pathways, we have summarized the therapeutic/diagnostic properties of ADCs bearing metal-based and nature-inspired payloads, as reported by the various authors. Most of the reviewed contributions highlighted that ADCs exhibit a more selective therapeutic activity towards cancer cells/tissues compared to non-conjugated payloads, as well as reduced side effects in animal models.

In summary, antibody–drug conjugates (ADCs) represent a rapidly evolving frontier in oncology and targeted therapy, with a future that promises significant advancements in cancer treatment. The innovative design of ADCs, which combines the specificity of monoclonal antibodies with the potent cytotoxicity of chemotherapy drugs, offers a targeted approach which can be used to destroy cancer cells while sparing healthy tissue. This precise mechanism of action has already shown impressive clinical efficacy in treating various malignancies, and ongoing research and development efforts suggest an even more transformative impact in the coming years. The future of ADCs looks particularly promising due to several key factors: (i) Advances in linker technology, which connects the antibody to the drug, are expected to enhance the stability and controlled release of the cytotoxic agent. Improved linkers will increase the therapeutic window and reduce off-target effects, making ADCs safer and more effective. (ii) Future ADCs will likely incorporate novel cytotoxic agents that are more potent and selective, enabling the treatment of tumors that are currently resistant to existing therapies. These new payloads could also reduce the required dosage, minimizing side effects. (iii) The identification of new cancer-specific antigens through genomic and proteomic technologies will allow for the development of ADCs targeting a broader range of cancers. This precision-medicine approach will enable tailored treatments for individual patients based on the unique molecular profile of their tumors. (iv) ADCs are expected to play a crucial role in combination therapies, working synergistically with other treatment modalities such as immune checkpoint inhibitors, CAR-T cells, and small molecule inhibitors, as well as bi- and multispecific antibodies. These combinations could potentiate the efficacy of each component, leading to more durable responses and potentially curative outcomes. (v) While the primary focus of ADCs has, so far, been oncology, there is growing interest in their application for other diseases, such as autoimmune disorders and infectious diseases. By leveraging the specificity of antibodies, ADCs could deliver therapeutic agents directly to affected cells, minimizing systemic toxicity and enhancing treatment efficacy. (vi) The integration of ADCs into personalized medicine strategies guided by biomarkers and diagnostic tools, will optimize patient selection and treatment planning. This approach ensures that patients receive the most appropriate and effective therapy based on their individual disease characteristics. 

We strongly believe that the future of antibody–drug conjugates is exceptionally bright, with the potential to revolutionize the landscape of cancer therapy and beyond. Ongoing research and clinical trials continue to expand our understanding and capabilities, paving the way for treatments which are more effective, safer, and personalized. As these innovations come to fruition, ADCs are poised to become a cornerstone of modern medicine, offering new hope to patients worldwide.
